# Acquisition of Resistance to RAS Inhibition Is Associated with the Upregulation of Macropinocytosis through Both PI3K-Dependent and -Independent Signaling

**DOI:** 10.1158/2767-9764.CRC-25-0731

**Published:** 2026-07-28

**Authors:** Ryan Robb, Sarah E. Ackermann, Seamus E. Degan, Khalilah E. Taylor, Runying Yang, Alexander Zuniga Marler, Valerie Calvert, Mariaelena Pierobon, Scott Lyons, Natalie Barker-Krantz, Aurora Cabrera, Antje Schaefer, Laura E. Herring, Adrienne D. Cox, Emanuel F. Petricoin, Clint A. Stalnecker, Kirsten L. Bryant

**Affiliations:** 1 Department of Pharmacology, University of North Carolina at Chapel Hill, Chapel Hill, North Carolina.; 2 Lineberger Comprehensive Cancer Center, University of North Carolina at Chapel Hill, Chapel Hill, North Carolina.; 3 Center for Applied Proteomics and Molecular Medicine, George Mason University, Fairfax, Virginia.; 4 Michael Hooker Proteomics Center, University of North Carolina at Chapel Hill, Chapel Hill, North Carolina.; 5 Department of Pharmacology and Toxicology, Medical College of Wisconsin, Milwaukee, Wisconsin.; 6 MCW Cancer Center, Medical College of Wisconsin, Milwaukee, Wisconsin.; 7 Department of Radiation Oncology, University of North Carolina at Chapel Hill, Chapel Hill, North Carolina.; 8 Department of Biochemistry and Biophysics, University of North Carolina at Chapel Hill, Chapel Hill, North Carolina.

## Abstract

Pancreatic ductal adenocarcinoma (PDAC) is a highly KRAS-addicted cancer. Although *KRAS*-mutant PDAC is susceptible to treatment with direct RAS inhibitors, drug resistance is an ongoing challenge. KRAS-driven PDAC is dependent on nutrient scavenging pathways such as macropinocytosis to fuel increased metabolic demands. Here, we report on the dynamics of macropinocytosis in response to genetic suppression and pharmacologic inhibition of KRAS and the RAS–ERK mitogen-activated protein kinase pathway. We show that macropinocytosis is transiently suppressed following acute genetic suppression of *KRAS* but is rapidly restored and even enhanced under prolonged RAS pathway inhibition. Furthermore, we demonstrate that human PDAC cell lines that have acquired resistance to RAS inhibitors, as well as cell lines derived from KPC tumors with acquired resistance to RAS inhibitors display markedly elevated macropinocytosis. RAS-inhibitor resistant cell lines also display increased abundance of macropinocytosis-related proteins and enhanced albumin uptake and sensitivity to albumin-bound paclitaxel. Mechanistically, this adaptive phenotype is mediated through both phosphoinositide 3-kinase (PI3K)-dependent and -independent pathways that converge on the activation of RAC1. Our results demonstrate a previously unrecognized link between RAS pathway inhibitor resistance and upregulation of macropinocytosis, underscoring the importance of further elucidating metabolic reprogramming in RASi-R PDAC.

**Significance:**

Macropinocytosis is upregulated following prolonged RAS-inhibitor treatment and in RASi-R models of PDAC via several heterogeneous mechanisms that converge on RAC1 activation and render RASi-R PDAC cell lines more sensitive to nab-paclitaxel.

## Introduction

Pancreatic cancer is currently the most lethal of all major types of cancer, with a 5-year survival rate of 13% (1). Pancreatic ductal adenocarcinoma (PDAC) is the most common form of pancreatic cancer, accounting for more than 90% of all cases (2). A defining feature of PDAC is the near-universal presence of activating mutations in the *KRAS* oncogene, which activate downstream signaling to drive tumor initiation and progression (3–5). Recent advances in chemical biology have resulted in the development of direct KRAS inhibitors (6–10). The two that are now FDA-approved are selective for the *KRAS*^*G12C*^ mutant (6–9), which is rare in PDAC (3, 11). However, *KRAS*^*G12D*^ mutations are present in >40% of patients with PDAC (3, 11), and KRAS^G12D^-selective inhibitors are under clinical assessment (10, 12). Furthermore, RAS(ON) multiselective RAS inhibitors that target all RAS isoforms and many different mutations are also under clinical evaluation (13–15). Daraxonrasib (RMC6236) has demonstrated clinical activity in previously treated advanced RAS-mutated pancreatic cancer (16) and is now being evaluated across PDAC disease settings, including first-line metastatic PDAC as monotherapy (17) or in combination with gemcitabine/nab-paclitaxel (18). These data have supported phase III evaluation, including RASolute 303 (NCT07491445), a global three-arm trial comparing daraxonrasib alone, daraxonrasib plus gemcitabine/nab-paclitaxel, and gemcitabine/nab-paclitaxel alone.

Although direct inhibitors of KRAS and RAS are displaying promising clinical efficacy in certain cancers, including *KRAS*-mutant PDAC, primary resistance occurs in more than half of patients, and acquired resistance to these agents invariably develops (3, 19). Therefore, uncovering and overcoming the mechanisms of RAS-inhibitor resistance is a current major focus of the PDAC research field. Resistance to RAS inhibitors can arise through a wide variety of adaptations (20–24), often those involving reactivation of RAS effector signaling or rewiring of parallel pathways (3, 25). In addition to its role in proliferative signaling, oncogenic KRAS drives metabolic reprogramming, enabling adaptation to the characteristically nutrient-poor PDAC tumor microenvironment (26–28). How such adaptive resistance mechanisms affect the metabolic processes necessary for tumorigenic growth and whether adaptations to RAS pathway inhibition at the metabolic level will reveal novel vulnerabilities are not currently understood.

One metabolic adaptation critical to the survival and growth of PDAC tumors is the induction of macropinocytosis (29–31), a form of fluid-phase endocytosis that allows cells to internalize large volumes of extracellular material to support anabolic metabolism (29–31). It is well-established that oncogenic RAS induces membrane ruffling (32), the first and critical step in macropinocytosis, and that oncogenic KRAS is a major driver of macropinocytosis in PDAC. Furthermore, macropinocytosis is elevated in *KRAS*-mutant PDAC and is thought to supply amino acids as well as other essential nutrients to the cancer cells (29–31). Although previous studies have demonstrated that macropinocytosis is acutely suppressed upon *KRAS* depletion (29, 33–35), the long-term dynamics of this process under sustained RAS pathway inhibition remain undefined. Furthermore, it is unclear whether resistant tumors maintain this suppression or evolve compensatory mechanisms to restore or enhance macropinocytic activity.

Here, we have examined the dynamics of macropinocytosis in *KRAS*-mutant PDAC cell lines upon genetic or pharmacologic suppression of KRAS signaling. We show that the downregulation of macropinocytosis upon genetic *KRAS* suppression is transient. Furthermore, we show that macropinocytosis is upregulated upon prolonged (7–18 days) pharmacologic inhibition of RAS, KRAS, or of the key RAS downstream effector pathway, the RAF–MEK–ERK mitogen-activated protein kinase (MAPK) kinase cascade. We found that acquired resistance to RAS inhibitors, in both human PDAC cell lines and lines derived from RAS inhibitor-resistant KPC tumors, is accompanied by enhanced levels of macropinocytosis. Also, we demonstrate that RAS inhibitor-resistant lines have elevated levels of proteins involved in the macropinocytotic machinery. Additionally, RAS inhibitor-resistant cell lines exhibit increased albumin uptake and sensitivity to albumin-bound paclitaxel (nab-paclitaxel). Mechanistically, RAS inhibitor-resistant lines exhibit increased levels of active RAC1. Upstream of RAC1 activation, we identify cell lines that rely on PI3K-, protein tyrosine kinase 2/focal adhesion kinase (PTK2/FAK), and TEA domain transcription factor (TEAD)-dependent signaling to mediate macropinocytosis. Our results demonstrate that nutrient scavenging is functionally altered in *KRAS*-mutant PDAC upon acquisition of RAS pathway inhibitor resistance, suggest possible future therapeutic directions, and illustrate the importance of identifying the full range of metabolic reprogramming upon pharmacologic KRAS inhibition.

## Materials and Methods

### Cell culture

Patient-derived xenograft (PDX) human *KRAS*-mutant PDAC cell lines (Pa14C and Pa16C) were provided by Dr. Anirban Maitra (MD Anderson Cancer Center). Established PDAC cell lines [HPAC (cat. #CRL-2119, RRID: CVCL_3517), MIA PaCa-2 (cat. #CRL-1420, RRID: CVCL_0428), SW1990 (cat. #CRL-2172, RRID: CVCL_1723), and PANC-1 (cat. #CRL-1469, RRID: CVCL_0480)] were obtained from the American Type Culture Collection. Cell lines derived from *Kras*^LSL-G12D/+^; *Trp53*^LSL-R172H/+^; *Pdx1-cre*^*tg/+*^ (KPC) tumors (K8484, K2293, K18745R, K18850R, K18399R, K18849R, and K18509R) were provided by Dr. Kenneth Olive (New York University). All lines were maintained in Dulbecco’s modified Eagle medium (DMEM, 11995065) or RPMI 1640 (11875093), supplemented with 10% fetal bovine serum (FBS), and kept in a humidified incubators at 37°C in 5% CO_2_.

Cell line identity was verified by short tandem repeat analysis for human PDAC cell lines, where applicable. All cell lines were tested for *Mycoplasma* using the MycoAlert Mycoplasma Detection Kit (Lonza). *Mycoplasma* testing was performed upon thawing each vial prior to experimental use, and all cell lines used in this study tested negative for *Mycoplasma*. Cells were cultured for no longer than 1 month after thawing before use in experiments, and new vials were thawed for subsequent experiments to minimize passage-associated drift.

### RAS inhibitor-resistant line generation

For each drug, early-passage parental cells were cultured, and 2 × 10^6^ cells were seeded in a 10-cm dish. The following day, cells were treated with the predetermined GI_50_ concentration for the parental cell line. Cells were allowed to grow to 90% confluency, and then 2 × 10^6^ viable cells (determined by trypan blue–free cell count) were seeded into two separate new 10-cm plates, with one plate treated with drug concentration escalated by a half log above the previous concentration and the other containing the previous concentration of drug to be used as a backup in case cells fail to grow in the escalated dose plate. This was repeated until treatment concentration reached 100× the original GI_50_ value. Once cells reached the final selection dose, cells were cultured and maintained in media containing drug at that concentration for at least 2 weeks prior to using them experiments. Cells were then maintained in final selection dose for all further culturing and experiments.

### Inhibitors

MRTX1133 (E1051) and MRTX849 (S8884) were purchased from SelleckChem. RMC7977 (HY-156498), trametinib (HY-10999), paclitaxel (HY-B0015), nab-paclitaxel (HY-P99974), pictilisib (HY-50094), defactinib (HY-12289), and IAG933 (HY-153811) were purchased from MedChemExpress.

### Antibodies

The following primary antibodies for immunoblotting were purchased from Cell Signaling Technology: p44/42 MAPK (MAPK3/MAPK1; ERK1/2; L34F12; cat. #4696; RRID: AB_390780), phospho-p44/42 MAPK (pERK1/2; Thr202/Tyr204; D13.14.4E; cat. #4370; RRID: AB_2315112), RSK1/RSK2/RSK3 (RPS6KA1/RPS6KA2/RPS6KA3; RSK; 32D7; cat. #9355; RRID: AB_659900), phospho-p90RSK (pRSK; Thr359/Ser363; cat. #9344; RRID: AB_331650), albumin (cat. #4929; RRID: AB_2225785), pan-AKT (AKT1/2/3; AKT; 40D4; cat. #2920; RRID: AB_1147620), phospho-AKT (Ser473; D9E; cat. #4060; RRID: AB_2315049), phospho-AKT (Tyr308; D25E6; cat. #13038; RRID: AB_2629447), GSK-3β (GSK3B; 27C10; cat. #9315; RRID: AB_490890), phospho-GSK-3β (Ser9; cat. #9336; AB_331405), mTOR (7C10; cat. #2983; AB_2105622), phospho-mTOR (Ser2448; D9C2; cat. #5536; RRID: AB_10691552), S6 ribosomal protein (RPS6; S6; 54D2; cat. #2317; RRID: AB_2238583), phospho-S6 ribosomal protein (pS6; Ser235/236; D57.2.2E; cat. #4858; RRID: AB_916156), paxillin (PXN; D9G12; cat. #12065; RRID: AB_2797814), YAP/TAZ (YAP1/WWTR1; D24E4; cat. #8418; RRID: AB_10950494), CYR61 (CCN1; E5W3H; cat. #39382; RRID: AB_2799154), and AXL receptor tyrosine kinase (AXL; C89E7; cat. #8661; RRID: AB_11217435), GAPDH (D16H11; cat. #5174; AB_10622025). The following primary antibodies for immunoblotting were purchased from Sigma-Aldrich: KRAS (cat. #WH0003845M1; RRID: AB_1842235) and vinculin (VCL; cat. #V9131; RRID: AB_477629). The following primary antibodies for immunoblotting were purchased from BD Transduction Laboratories: RAC1 (cat. #610650; RRID: AB_397978) and FAK (cat. #610087; RRID: AB_397494). The following primary antibodies for immunoblotting were purchased from Invitrogen: phospho-FAK (Tyr397; 31H5L17; cat. #700255; RRID: AB_397494) and phospho-paxillin (Tyr118; cat. #44-722G; RRID: AB_2533733). Primary antibody active YAP1 (EPR19812; cat. #ab205270; RRID: AB_2813833) for immunoblotting was purchased from Abcam. Secondary antibodies used for immunoblotting included IgG (H + L) cross-adsorbed goat anti-mouse, horseradish peroxidase (HRP), Invitrogen (cat. #31432; RRID: AB_228302), IgG (H + L) cross-adsorbed goat anti-rabbit, HRP, Invitrogen (cat. #31462; RRID: AB_228338), IRDye 800CW goat anti-rabbit IgG secondary antibody LICORbio (cat. #926-32211; RRID: AB_621843), and IRDye 680LT donkey anti-mouse IgG secondary antibody LICORbio (cat. #926-68022; RRID: AB_10715072). All primary and secondary antibodies were used at concentrations following the manufacturer’s instructions.

### siRNA transfections

For siRNA transfections, cells were seeded and incubated for 24 hours at 37°C. The following day, transfection complexes were prepared using Opti-MEM I (Life Technologies, #31985-070), Lipofectamine RNAiMAX (Life Technologies, #13778-150), and Silencer Select siRNA (Thermo Fisher Scientific; target-specific), following the ratios and method recommended in the manufacturer’s protocol. To prepare the transfection mixture, Lipofectamine RNAiMAX was added to Opti-MEM and gently mixed by pipetting. Separately, siRNA was diluted in Opti-MEM. The two solutions were then combined at a 1:1 ratio, mixed gently, and incubated at room temperature for 30 minutes to allow complex formation. After incubation, the siRNA–Lipofectamine complex was added in a dropwise manner to each well containing cells, and the plate was gently rocked to distribute the mixture evenly. Transfected cells were incubated for 24, 72, 120, or 168 hours at 37°C, after which they were processed for respective assays.

### Immunoblotting

Cells were washed twice on ice with PBS and lysed using RIPA lysis buffer (Pierce; 89900) supplemented with phosphatase inhibitor cocktails I and II (Millipore; 524624 and 524625) and a protease inhibitor cocktail (Roche; 11873580001). Lysates were collected by scraping cells into microcentrifuge tubes, homogenized via sonication, and centrifuged at >15,000 × *g* for 10 minutes at 4°C to pellet. The supernatant was transferred to a new microcentrifuge tube, and protein concentrations were measured using the Bradford assay (Bio-Rad; 5000006) or DC protein assay (Bio-Rad; 5000112). A measure of 20 to 30 μg of protein were mixed with 4× Laemmli sample buffer containing a reducing agent (either DTT or β-mercaptoethanol) and boiled for 5 minutes at 95°C. Samples were then resolved by SDS-PAGE and transferred to polyvinylidene difluoride membranes. Membranes were blocked for 1 hour in 5% (w/v) bovine serum albumin (BSA) in TBS with 0.05% Tween-20 (TBST) at room temperature, followed by incubation in primary antibodies overnight at 4°C. Membranes were washed three times in TBST for 10 minutes each then incubated with secondary antibodies (1:5,000 dilution) for 1 hour. Membranes were washed again three times in TBST for 10 minutes, and protein bands were visualized by chemiluminescence or fluorescence (LICORbio).

### Cell viability

Cells were seeded into flat, clear-bottom 96-well plates (Corning) at a density of 750 to 1,500 cells per well. A separate plate designated as the day 0 reference was prepared in parallel. All plates were incubated for 24 hours at 37°C in 5% CO_2_. After incubation, treatment plates received drug compounds via the Tecan D300e Digital Dispenser and were placed back in incubator, and the day 0 plate was stained with Hoechst (final concentration 3 μmol/L) for 30 minutes to resolve nuclei. Day 0 nuclei were imaged, and cell count was measured using the BioTek Cytation 1 Cell Imaging Multi-Mode Reader. For *KRAS* knockdown assays, siRNA-transfected plates were incubated for an additional 1 to 7 days after transfection. For drug dose–response assays, treatment plates were incubated for an additional 5 days after treatment. At the respective time points, cells were labeled with Hoechst, and cell counts were measured using the same procedure as on day 0.

### Colony formation

For long-term colony formation growth assays, 1,000 cells were seeded in six-cm dishes and allowed to adhere overnight. Cells were treated according to experimental conditions the following day as indicated, and drug media were refreshed every 3 days. After 12 to 16 days depending on the cell line, cells were fixed with a methanol-based fixation solution (glacial acetic acid: methanol in a 1:7 ratio, vol/vol). Media and fixative were then removed, dishes were rinsed by gently dunking in water, and cells were stained with 2 mL of 1% crystal violet in ddH_2_O for 15 minutes. Following staining, the solution was removed, and dishes were gently rinsed with water until no residual dye remained and allowed to dry. Imaging was performed using a Typhoon FLA 9500 scanner with Alexa Fluor 647 settings to detect crystal violet–stained colonies. Images were processed in FIJI, where a fluorescence threshold was applied to quantify the stained area by calculating the percent area covered per dish.

### Flow cytometry macropinocytic uptake assay

Cells were seeded into 12-well plates (Corning) and treated according to experimental conditions the following day as indicated. One hour prior to harvesting cells for analysis, cells were washed with PBS, and media were replaced with 0.5 mg/mL working solution of tetramethylrhodamine (TMR)-conjugated 70 kD dextran or TMR-BSA in DMEM without FBS and incubated 1 hour at 37°C. Cells were then washed three times with PBS, trypsinized, and collected into microcentrifuge tubes and pelleted via centrifugation at 400 × *g* for 5 minutes. Cells were fixed by resuspending in 250 μL of 2% paraformaldehyde in PBS for 15 minutes at room temperature and then washed with 1 mL PBS. Finally, cells were pelleted again at 400 × *g* for 5 minutes and resuspended in PBS. Cells were then kept on ice, and fluorescence intensity was analyzed via flow cytometry. All steps were performed in the dark to protect fluorescent signal.

### Fluorescence microscopy macropinocytosis assay

Cells were seeded into flat, glass bottom 12-well plates (MatTek) and treated according to experimental conditions the following day as indicated. Cells were washed with PBS, and media were replaced with 1 mg/mL working solution of FITC-conjugated 70 kD dextran in DMEM without FBS and incubated 30 minutes at 37°C prior to the indicated time points. Cells were washed five times with ice-cold PBS to remove residual dextran then fixed in 4% paraformaldehyde for 30 minutes at room temperature. After fixation, cells were washed three times with PBS and then incubated in 2 μmol/L Hoechst in PBS to stain nuclei for 30 minutes at room temperature. Cells were washed three times with PBS, and then 1 mL pH-adjusted antifade fluorescence imaging media (50% glycerol + 0.5% N-propyl gallate in PBS) was added to each well. Images were acquired on an EVOS M7000 Imaging System using phase-contrast, 4′,6-diamidino-2-phenylindole (DAPI), and GFP channels with a 40× objective. FITC-labeled area was quantified using thresholding and particle analysis in ImageJ as previously described. Quantification of average uptake of FITC–dextran or FITC–BSA per cell was calculated as the FITC–dextran- or FITC–BSA-labeled area divided by cell count in each imaged field.

### Reverse-phase protein array

Cells were plated into six-well dishes (Corning) and treated with trametinib, MRTX 1133, or RMC7977 for 24, 72, 120, or 168 hours. At the respective time point, samples were washed with PBS, snap-frozen on liquid nitrogen, and stored at −80°C until all samples were collected. All treatments were done in biological quadruplets.

Lysates were prepared and loaded in triplicate (approx. 10 nL per spot using a Quanterix 2470 Arrayer (Quanterix) onto nitrocellulose-coated slides (Grace Biolabs). Standard curves of control cell lysates were included. A total of 191 antibodies were used in reverse-phase protein array (RPPA) experiments (Supplementary Table S1). Antibodies were confirmed to have a single band at the appropriate molecular weight before inclusion. Each slide was immunostained with a single primary antibody. For secondary antibodies, either biotinylated goat anti-rabbit IgG (H + L; 1:7,500, Vector Laboratories) or rabbit anti-mouse IgG (1:10, DakoCytomation) was used. The signal amplification process was performed using a tyramide-based avidin/biotin amplification system (DakoCytomation) followed by streptavidin-conjugated IRDye 680 (LICORbio). Secondary antibody alone was used as a negative control. A total protein blot was performed using SpyroRed as per the manufacturer’s instructions (Molecular Probes). Data were collected directly from images visualized using a Tecan PowerScanner (Tecan) and measured with MicroVigene software version 5.1.0.0 (Vigenetech). For each sample, the total protein levels were calculated via an averaging of SpyroRed dot intensity for each replicate. Negative control spot intensities were subtracted from primary antibody spot intensities, averaged across triplicate spots, and normalized to total protein intensity per sample to generate the final intensity. Principal component analysis (PCA) was conducted to evaluate the grouping of replicates, treatment conditions, time points, and cell lines. All samples were retained for subsequent analysis. Antibody signals that were either undetected or had intensity values below one were excluded on a per-sample basis. Signal intensities were transformed using a log_2_ scale, normalized to the median, and analyzed using the LIMMA package (version 3.56.2) to identify differential expression and statistical significance through linear modeling and empirical Bayes moderation. All data analysis was carried out in R (version 4.5.1).

### Total proteomics preprocessing and analysis

#### Sample preparation

PANC-1 cells were plated and treated with DMSO or RASi (RMC7977; 24 or 168 hours), and additional sample was collected from a RAS inhibitor-resistant PANC-1 model. Cells were snap-frozen and lysed [Thermo Lysis Solution (cat. #1902574) with Halt Protease and Phosphatase Inhibitor Cocktail (cat. #78440)]. Lysates were collected, sonicated (20 seconds), and centrifuged at maximum speed for 15 minutes at 4°C to generate a purified lysate. Protein (75 μg per sample) was prepared with the AccelerOme System (Thermo Fisher) using a label-free AccelerOme sample preparation kit (cat. #A50945) according to the manufacturer’s protocol. The built-in label-free method was used to perform reduction, alkylation, LysC/trypsin digestion, and peptide clean-up. Peptide concentration was measured using an in-line UV spectrophotometer. All samples were dried down via lyophilization and stored at −80°C prior to LC/MS-MS analysis.

#### LC/MS-MS analysis

All samples were reconstituted in 2% ACN in 0.1% formic acid and normalized to 0.1 μg/μL. Samples were analyzed via LC/MS-MS using a Vanquish Neo coupled to an Orbitrap Astral mass spectrometer (Thermo Fisher Scientific). A pooled sample was created by combining an aliquot of each sample, which was analyzed intermittently throughout the sequence to assess technical reproducibility. Samples (2 μL) were injected onto an Aurora Ultimate TS column (75 μm id × 25 cm, 1.7 μm particle size; IonOpticks) and separated over a 30-minute method. The gradient consisted of 5% to 45% mobile phase B at a 300 nL/minute flow rate, in which mobile phase A was 0.1% formic acid in water and mobile phase B was 0.1% formic acid in 80% acetonitrile. MS1 full scans (m/z 350–980) were acquired with a resolution of 240,000 in the Orbitrap analyzer with a maximum injection time of 5 milliseconds and an AGC target of 500%. MS2 scans were acquired using the Orbitrap Astral detector operated in data-independent acquisition mode, covering a scan range of m/z 150 to 2,000 with a cycle time of 0.6 seconds. Isolation windows were set to 3 m/z with a higher collision dissociation energy of 25%, an AGC target of 500%, and a maximum injection time of 3 milliseconds.

#### Data analysis

Raw data files were processed using Spectronaut (v19; Biognosys) and searched against the UniProt reviewed human database (UP000005640, containing 20,434 entries, downloaded January 2025) and the MaxQuant common contaminants database (246 entries). The following settings were used: enzyme specificity set to trypsin, up to two missed cleavages allowed, cysteine carbamidomethylation set as a fixed modification, and methionine oxidation and N-terminal acetylation set as variable modifications. A false discovery rate (FDR) of 1% was used to filter all data. Imputation was disabled, and single-hit proteins were retained. Differential expression analysis was performed in R (v4.5.1) using LIMMA (v3.56.2). Proteins were normalized by total intensity for each sample before performing differential expression analysis and empirical Bayes moderation in LIMMA. Unpaired Student *t* tests were conducted, and *P* values, FDR-corrected *P* values (q values), along with log_2_ fold change (FC) ratios were calculated in Spectronaut. Proteins with an absolute log_2_ FC ≥0.5 and a *q* value (corrected *P* value) <0.05 were considered significant.

### Statistical analysis

All statistical analyses, unless otherwise stated, were conducted using GraphPad Prism version 10.4.1, with specific tests detailed in the respective figure legends. Unpaired *t* tests were used to compare a single treatment with control, and all data are shown relative to their appropriate control groups. Unless otherwise noted, error bars on all graphs represent either the mean or median ± standard error of the mean (SEM.) from at least three independent biological experiments (*n* ≥ 3) or standard deviation (SD) if presented as one representative experiment to show spread of data (e.g., macropinocytic index). Correlation coefficients (r values) are displayed within each figure panel. The number of samples included in each experiment, along with clarification on whether the data reflect pooled results from multiple experiments or a single representative trial, is provided in the figure legends.

Quantification of colony formation was performed in FIJI by calculating the percent area covered by crystal violet staining in each well. To do this, a binary mask was created to isolate the stained regions, and this mask was then used to measure the proportion of the total well area occupied by colonies. Percent coverage values were normalized to the vehicle-treated control well, which was designated as 100% for each biological replicate. The mean and SD were calculated for each replicate group, and data were plotted against the logarithm of drug concentration using GraphPad Prism version 10.4.1. Dose–response curves were generated using a four-parameter (variable slope) nonlinear regression model based on the following equation: Y = bottom + (top–bottom)/[1 + 10^((LogIC50-X) × HillSlope)^].

## Results

### Downregulation of macropinocytosis in *KRAS*-mutant PDAC cell lines is not sustained following genetic loss of *KRAS*

**Figure 1.**
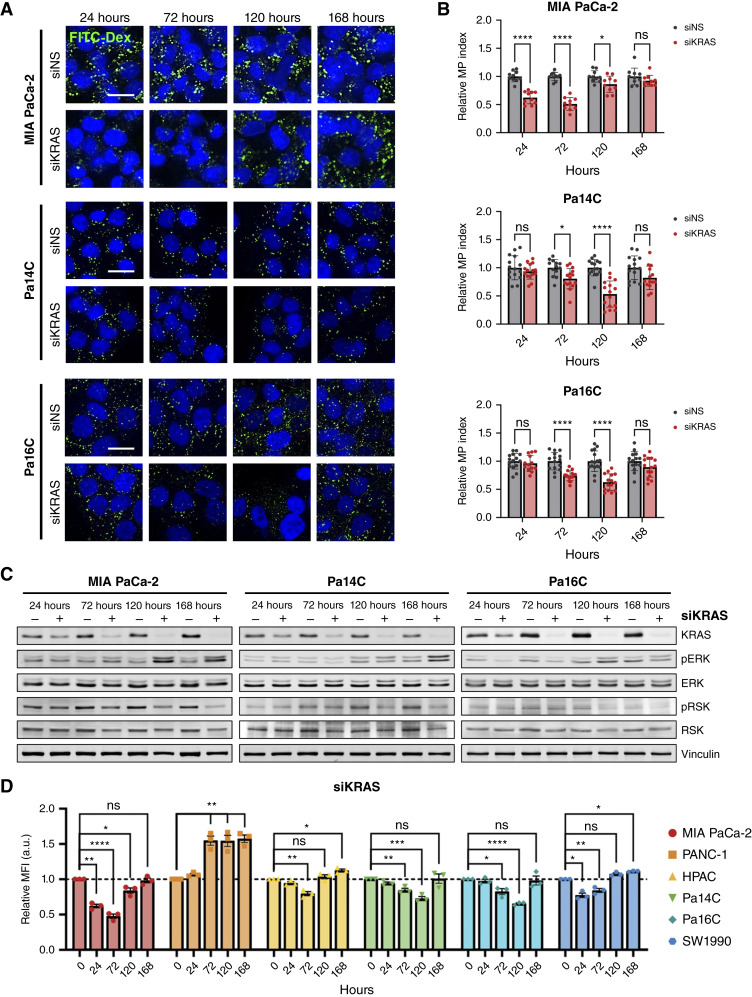
Downregulation of macropinocytosis is not sustained following genetic loss of *KRAS* in *KRAS*-mutant PDAC cell lines. **A,** Representative images of macropinosomes labeled with FITC–dextran (green) and nuclear DAPI stain (blue) in indicated *KRAS*-mutant PDAC cells transiently transfected with an siRNA oligonucleotide (10 nmol/L) against *KRAS* (siKRAS) or a nonspecific control (siNS) for indicated time points. Images are representative of 10 fields of view analyzed in each of three independent experiments. Scale bars, 20 μm. **B,** Quantification of (**A**), in which the total area of macropinosomes (FITC–dextran immunofluorescence) was calculated and normalized to cell number [macropinocytic (MP) index]. The relative MP index is plotted, with each individual data point representing one field containing at least 10 analyzed cells. Data for MIA PaCa-2, Pa14C, and Pa16C are presented as the mean ± SD of one experiment that is representative of three independent experiments. *, ρ < 0.05 and ****, ρ < 0.0001, by the unpaired Student *t* test, comparing against NS. **C,** Immunoblots of indicated *KRAS*-mutant PDAC cell lines treated with siNS and siKRAS as in **A**. Vinculin levels were used to monitor equivalent total protein loading. Blots are representative of three independent experiments. **D,** Macropinocytosis was measured via flow cytometry in *KRAS*-mutant PDAC cell lines that were treated with siRNA oligonucleotides as in **A**. Macropinocytosis was quantified via TMR–dextran labeling. Data are presented as the mean ± SEM of three independent experiments. *, ρ < 0.05; **, ρ < 0.01; ***, ρ < 0.001, and ****, ρ < 0.0001, by the unpaired Student *t* test, comparing against NS. Dex, dextran; MFI, median fluorescence intensity; ns, not significant.

Previous studies have shown that genetic suppression of *KRAS* results in downregulation of macropinocytosis after 24 to 72 hours of RNAi treatment (29, 33–35). To determine whether this downregulation is sustained following prolonged depletion of oncogenic KRAS, we assessed macropinocytic uptake in a panel of *KRAS*-mutant PDAC cell lines after as long as 168 hours of siRNA treatment (Fig. 1A; Supplementary Fig. S1A). Macropinosomes were visualized via fluorescence microscopy based on the ability of cells to internalize extracellular medium containing fluorescein isothiocyanate-labeled 70 kDa dextran (FITC-Dex), an established marker of macropinosomes (36). In agreement with previous findings (29, 33–35), we observed a significant reduction in macropinocytosis within 72 hours; however, by 168 hours, macropinocytosis returned to basal levels (Fig. 1B) or nearly so (Supplementary Fig. S1B). We confirmed via immunoblot analysis that KRAS suppression was maintained over the duration of the 168-hour time-course (Fig. 1C; Supplementary Fig. S1C) and that KRAS suppression led to a decrease in cell proliferation (Supplementary Fig. S1D). We hypothesized that the return of macropinocytosis to basal levels may correlate with activation of canonical RAS signaling through the ERK MAPK or PI3K–AKT pathway. To assess this hypothesis, we performed immunoblots for phosphorylated and total ERK, RSK, and AKT (Fig. 1C; Supplementary Fig. S1C and S1E). Although activation of these proteins was observed in subsets of the cell lines over time, there was not one marker that correlated with increased macropinocytosis, leading us to conclude that additional pathways must be mediating macropinocytic upregulation.

As a complementary approach to fluorescence microscopy, we utilized flow cytometry to measure TMR-labeled 70 kDa dextran (TMR–dextran) uptake (37) under identical experimental conditions. Again, we found that macropinocytosis returned to or surpassed basal levels by 168 hours (Fig. 1D). We conclude that the decrease in macropinocytosis that occurs upon genetic suppression of KRAS is transient, returning to or surpassing basal levels via one or more KRAS-independent mechanisms.

### Prolonged pharmacologic inhibition of RAS or the RAS–ERK MAPK effector pathway upregulates macropinocytosis

Having demonstrated that macropinocytosis was downregulated only transiently following genetic suppression of *KRAS*, we next evaluated whether pharmacologic inhibition of RAS would have a similar effect. For our panel of *KRAS*-mutant PDAC cell lines, we purposely selected lines which exhibit a wide range of sensitivity to RAS and ERK MAPK pathway inhibition (Supplementary Fig. S2A). To maintain consistency throughout our panel of cell lines, we treated cells at their respective approximate GI_60_ doses to each inhibitor (Supplementary Fig. S2A) for all subsequent time-course experiments.

**Figure 2.**
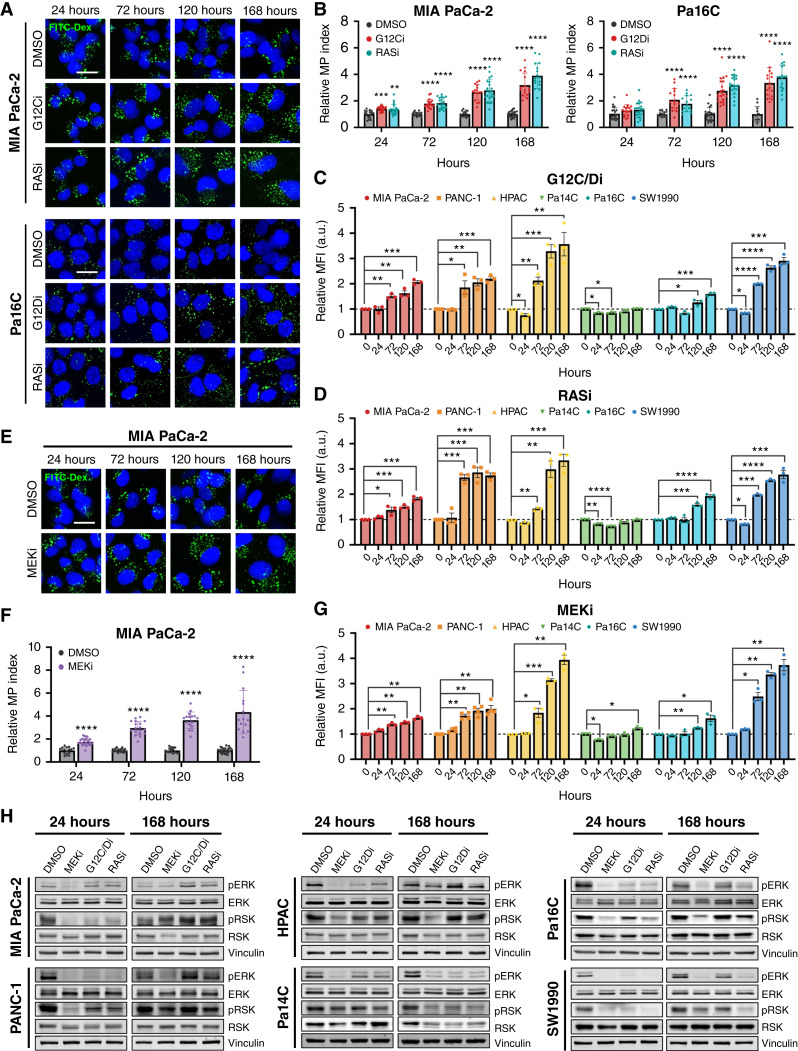
Prolonged inhibition of the RAS ERK–MAPK pathway upregulates macropinocytosis. **A,** Representative images of macropinosomes labeled with FITC–dextran (green) and nuclear DAPI stain (blue) in indicated *KRAS*-mutant PDAC cells treated with DMSO or a GI_60_ dose of MRTX849 (G12Ci), MRTX1133 (G12Di), or RMC7977 (RASi). Images are representative of 10 fields of view analyzed in each of three independent experiments. Scale bars, 20 μm. **B,** Quantification of (**A**), in which the relative macropinocytic (MP) index is plotted, with each individual data point representing one field containing at least 10 analyzed cells. Data for MIA PaCa-2 and Pa16C are presented as the mean ± SD of one experiment that is representative of three independent experiments. **, ρ < 0.01; ***, ρ < 0.001; and ****, ρ < 0.0001, by the unpaired Student *t* test, comparing against DMSO. **C** and **D,** Macropinocytosis was measured via flow cytometry in *KRAS*-mutant PDAC cell lines that were treated with a GI_60_ dose of (**C**) MRTX849 (G12Ci) or MRTX1133 (G12Di) and (**D**) RMC7977 (RASi). Macropinocytosis was quantified via TMR–dextran labeling. Data are presented as the mean ± SEM of three independent experiments. *, ρ < 0.05; **, ρ < 0.01; ***, ρ < 0.001, and ****, ρ < 0.0001, by the unpaired Student *t* test, comparing against DMSO. **E,** Representative images of macropinosomes labeled with FITC–dextran (green) and nuclear DAPI stain (blue) in MIA PaCa-2 cells treated with DMSO or a GI_60_ dose of trametinib (MEKi). Images are representative of 10 fields of view analyzed in each of three independent experiments. Scale bars, 20 μm. **F,** Quantification of (**E**), in which the relative MP index is plotted, with each individual data point representing one field containing at least 10 analyzed cells. Data for MIA PaCa-2 and are presented as the mean ± SD of one experiment that is representative of three independent experiments. ****, ρ < 0.0001, by the unpaired Student *t* test, comparing against DMSO. **G,** Macropinocytosis was measured via flow cytometry in *KRAS*-mutant PDAC cell lines that were treated with a GI_60_ dose of trametinib (MEKi). Macropinocytosis was quantified via TMR–dextran labeling. Data are presented as the mean ± SEM of three independent experiments. *, ρ < 0.05; **, ρ < 0.01; ***, ρ < 0.001, and ****, ρ < 0.0001, by the unpaired Student *t* test, comparing against DMSO. **H,** Immunoblots of indicated KRAS-mutant PDAC cell lines treated DMSO or a GI_60_ dose of trametinib (MEKi), MRTX849 (G12Ci), MRTX1133 (G12Di), or RMC7977 (RASi). Vinculin levels were used to monitor equivalent total protein loading. Blots are representative of three independent experiments. Dex, dextran; MFI, median fluorescence intensity.

To assess the regulation of macropinocytic uptake following pharmacologic inhibition of RAS, we first treated *KRAS*-mutant cell lines for 24 to 168 hours with either the mutant-selective KRAS inhibitor MRTX849 (G12Ci; ref. 38) for *KRAS*^*G12C-*^mutant cells or MRTX1133 (G12Di; ref. 12) for *KRAS*^*G12D-*^mutant cells, or with a RAS(ON) multiselective inhibitor (RMC7977; RASi; ref. 13) for both, and measured macropinocytosis by FITC–dextran uptake via fluorescence microscopy (Fig. 2A; Supplementary Fig. S2B). In contrast to the effect of genetic depletion of *KRAS*, we did not observe substantial downregulation of macropinocytosis following 24-hour treatment with G12C/Di or RASi. However, we did observe a significant increase in macropinocytosis by 72 hours (Fig. 2B; Supplementary Fig. S2C).

As an orthogonal approach, we measured macropinocytic uptake of TMR–dextran via flow cytometry following treatment with G12C/Di or RASi (Fig. 2C and D). The results showed a significant increase in macropinocytic uptake by 168 hours in five of the six cell lines tested. Additionally, when we extended the time-course to 18 days, we observed that macropinocytosis remained elevated or increased even further compared with 168 hours (7 days) of treatment (Supplementary Fig. S2D). Furthermore, the one cell line (Pa14C) that did not display an increase by 168 hours did exhibit a significant increase at 18 days of treatment. We conclude that treatment with direct inhibitors of RAS increases macropinocytosis in *KRAS*-mutant PDAC cell lines.

In PDAC, the major effector pathway downstream of KRAS is the ERK–MAPK pathway, comprised of the RAF, MEK, and ERK kinases (3–5). We therefore tested whether inhibiting the downstream MAPK pathway proteins, MAP2K1 and MAP2K2 (MEK1/2; hereafter referred to as MEK), would also increase macropinocytosis. We measured macropinocytosis via microscopy following treatment with trametinib [MEK inhibitor (MEKi); Fig. 2E; Supplementary Fig. S2E]. Similar to G12C/Di or RASi treatment, we observed a significant increase in macropinocytosis following MEKi treatment in all four cell lines tested (Fig. 2F; Supplementary Fig. S2F). The timing and magnitude of MEKi-induced macropinocytosis were cell line–dependent. We next measured macropinocytosis via flow cytometry following treatment with either MEKi or SCH772984 (ERKi; Fig. 2G; Supplementary Fig. S2G). Similar to the results with direct RAS inhibitors, we observed a robust increase in macropinocytosis over the duration of both MEK and ERK inhibition. Consistent with previous studies (38, 39), we observed transient loss of MEK targets pERK and pRSK (Fig. 2H). Together, these data indicate that PDAC cells upregulate macropinocytosis in response to long-term inhibition of the RAS ERK–MAPK pathway.

### PDAC cell lines with acquired resistance to pharmacologic inhibitors of RAS also exhibit enhanced macropinocytosis

**Figure 3.**
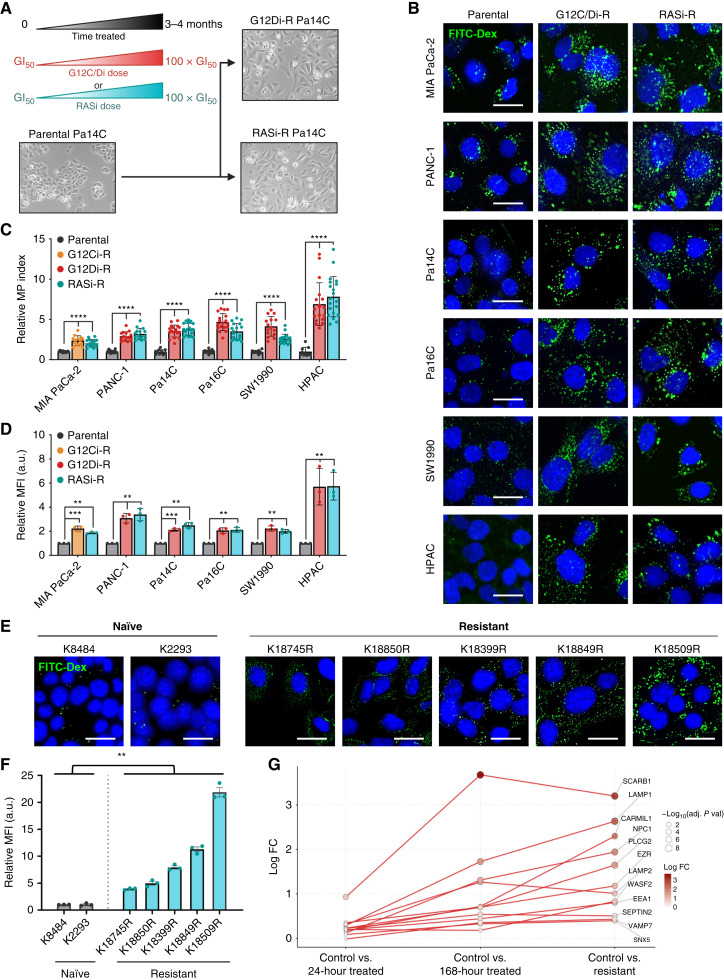
PDAC cell lines with acquired resistance to pharmacologic inhibitors of RAS exhibit enhanced macropinocytosis. **A,** Schematic diagram showing the experimental design for generating cell lines with acquired resistance to MRTX1133 (G12Di) and RMC7797 (RASi). **B,** Representative images of macropinosomes labeled with FITC–dextran (green) and nuclear DAPI stain (blue) in indicated parental or MRTX849- (G12Ci-R), MRTX1133- (G12Di-R), or RMC7797-resistant (RASi-R) *KRAS*-mutant PDAC cells. Images are representative of 10 fields of view analyzed in each of three independent experiments. Scale bars, 20 μm. **C,** Quantification of relative macropinocytic (MP) index, with each individual data point representing one field containing at least 10 analyzed cells. Data are presented as the mean ± SD of one experiment that is representative of three independent experiments. ****, ρ < 0.0001, by the unpaired Student *t* test, comparing against the respective parental cell line. **D,** Macropinocytic uptake of TMR–dextran was measured by MFI via flow cytometry in indicated parental or MRTX849- (G12Ci-R), MRTX1133- (G12Di-R), or RMC7797-resistant (RASi-R) *KRAS*-mutant PDAC cell lines. Data are presented as the mean ± SEM of three independent experiments. **, ρ < 0.01 and ***, ρ < 0.001 by the unpaired Student *t* test, comparing against the respective parental cell line. **E,** Representative images of macropinosomes labeled with FITC–dextran (green) and nuclear DAPI stain (blue) in indicated treatment-naïve (naïve) or RMC7797-resistant (resistant) tumor-derived KPC cells. Images are representative of 10 fields of view analyzed in each of three independent experiments. Scale bars, 20 μm. **F,** Macropinocytic uptake of TMR–dextran was measured by MFI via flow cytometry in indicated treatment-naïve (naïve) or RMC7797-resistant (resistant) tumor-derived KPC cell lines. Data are presented as the mean ± SEM of three independent experiments. **, ρ < 0.01 by nested two-way ANOVA. **G,** Relative levels of selected macropinocytosis-related proteins in PANC-1 cells following 24 or 168 hours of RASi treatment, as well as in a RASi-R cell line. Line color represents the direction of change from 24 hours RASi treatment to RASi resistance, and line transparency represents the magnitude of change. Dex, dextran; MFI, median fluorescence intensity. [**A,** Created in BioRender. Robb, R. (2026) https://BioRender.com/rx7i90i.]

Because we saw a consistent and sustained increase in macropinocytosis following prolonged RAS inhibition, we next sought to determine whether PDAC cells would maintain high macropinocytic activity upon acquiring resistance to RAS-inhibitor treatment. We generated a panel of G12C/Di-resistant (G12C/Di-R) and RASi-resistant (RASi-R) cell lines via dose escalation over time (Fig. 3A). Briefly, we began selection by treating cells with approximately GI_50_ doses and allowed them to grow to confluency. We then replated the cells, increasing the treatment concentration 2- to 3-fold, and repeated this cycle until the cells tolerated ∼100-fold of the original GI_50_ dose. G12C/Di-R and RASi-R cell lines were maintained and tested in the final selection dose for all subsequent experiments. Resistance to the corresponding inhibitor used for selection was validated via 5-day dose–response proliferation assays performed in parallel, comparing G12C/Di-R or RASi-R lines with parental lines (Supplementary Fig. S3A). We also observed robust morphologic changes between the parental and resistant cell lines, with the resistant cell lines exhibiting a consistently larger and flatter morphology than their parental counterparts (Supplementary Fig. S3B).

Next, we assessed macropinocytic uptake in G12C/Di-R or RASi-R lines compared with parental lines. Quantification of FITC–dextran-labeled macropinosomes revealed that all G12C/Di-R and RASi-R lines displayed strikingly higher levels of macropinocytosis, ranging from 2- to 8-fold greater than their respective parental lines (Fig. 3B and C). These results were recapitulated via flow cytometry analysis of TMR–dextran uptake, measured by fluorescence intensity (Fig. 3D).

We next sought to determine whether *in vivo* development of RAS-inhibitor resistance results in upregulation of macropinocytosis, consistent with our observation in *in vitro*–generated resistant PDAC lines. To test this, we assessed macropinocytosis in a panel of cell lines derived from endpoint KPC mouse pancreatic tumors treated with either vehicle (treatment-naïve) or continuous RASi treatment following relapse and outgrowth (resistant; ref. 14). Resistant lines were cultured and maintained in media containing 10 nmol/L RASi (∼10-fold IC_50_ of treatment-naïve lines). Fluorescence microscopy imaging of FITC–dextran-labeled macropinosomes clearly demonstrated that all resistant lines exhibited higher macropinocytosis than treatment-naïve cells (Fig. 3E). Flow cytometry analysis revealed that macropinocytic uptake across the panel of resistant lines ranged from ∼4- to 22-fold greater than treatment-naïve lines (Fig. 3F).

Having observed that macropinocytic activity increased over time and upon acquisition of resistance to RAS inhibition, we next investigated changes in expression of macropinocytosis-related proteins. We performed total proteomics in parental and RASi-R PANC-1 cells treated with RASi for 24 and 168 hours. We observed dynamic changes in the proteome that increased with treatment time and with the acquisition of RASi resistance (Supplementary Fig. S4A and S4B). In particular, we found that expression of critical proteins involved in cargo uptake (e.g., CARMIL1, SCARB1, WASF2, and NPC1), macropinosome maturation (e.g., EEA1, SNX, and Septins), and cargo degradation (e.g., SNAREs and LAMP1/2) increased over time and were maintained upon acquisition of resistance (Fig. 3G). We conclude that RASi-R PDAC cells maintain upregulated levels of macropinocytosis by increasing the protein abundance of the macropinocytic machinery.

### Upregulated macropinocytosis in RAS inhibitor-resistant cell lines is associated with increased uptake of and sensitivity to albumin-bound paclitaxel compared with free drug

**Figure 4.**
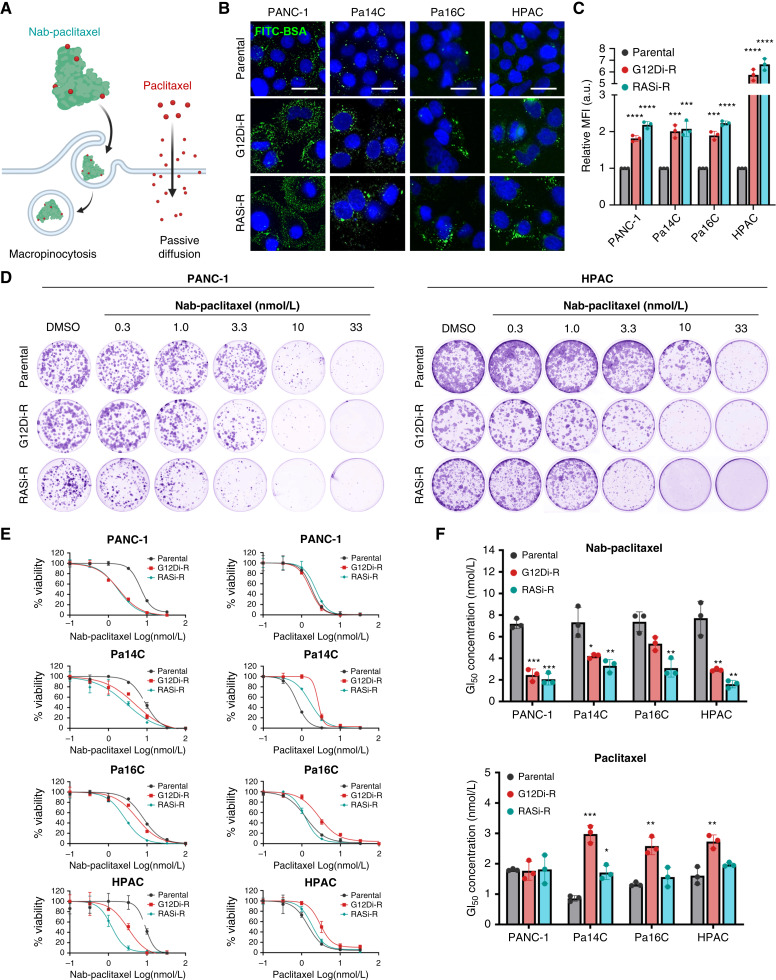
Upregulated macropinocytosis in RAS inhibitor-resistant cell lines is associated with increased uptake of and sensitivity to albumin-bound paclitaxel compared with free drug. **A,** Schematic diagram highlighting the differential routes through which free paclitaxel and albumin-bound nab-paclitaxel enter the cell. **B,** Representative images of macropinosomes labeled with FITC-BSA (green) and nuclear DAPI stain (blue) in indicated parental or MRTX1133- (G12Di-R) or RMC7797-resistant (RASi-R) *KRAS*-mutant PDAC cells. Images are representative of 10 fields of view analyzed in each of two independent experiments. Scale bars, 20 μm. **C,** Quantification of median intensity of TMR-BSA fluorescence in indicated MRTX1133- (G12Di-R) or RMC7797-resistant (RASi-R) *KRAS*-mutant PDAC cell lines plotted relative to matched parental cell line, with each individual data point representing one field containing at least 10 analyzed cells. Data are presented as the mean ± SEM of three independent experiments. ***, ρ < 0.001, and ****, ρ < 0.0001, by the unpaired Student *t* test, comparing against the parental cell line. **D,** Representative images of clonogenic growth assays following treatment of matched parental and either MRTX1133- (G12Di-R) or RMC7977-resistant (RASi-R) PANC-1 or HPAC cell lines treated with nab-paclitaxel for 12–16 days. **E,** Relative colony number of matched parental and either MRTX1133- (G12Di-R) or RMC7977-resistant (RASi-R) PDAC cell lines treated with nab-paclitaxel (left) or paclitaxel (right) for 12–16 days. Data are presented as the mean ± SEM of three independent experiments. **F,** GI_50_ values from data plotted in (**E**) for nab-paclitaxel treated lines (top) and paclitaxel treated lines (bottom). *, ρ < 0.05; **, ρ < 0.01; ***, ρ < 0.001, by the unpaired Student *t* test, comparing against the parental cell line. [**A,** Created in BioRender. Robb, R. (2026) https://BioRender.com/nyleshf.]

Drug delivery systems utilizing large carriers, such as albumin, micelles, liposomes, and exosomes, have been shown to undergo active internalization by cancer cells via macropinocytosis, indicating that enhancing the macropinocytic pathway could improve anticancer drug delivery (40, 41). Notably, previous studies have shown that macropinocytosis is the dominant mechanism responsible for uptake of albumin-bound drugs (40, 42) such as nab-paclitaxel, an albumin-bound standard-of-care therapeutic for *KRAS*-mutant PDAC (43). Conversely, free paclitaxel is cell membrane–permeable, entering the cell through passive diffusion (Fig. 4A), and is not a standard-of-care in PDAC. The upregulation of macropinocytosis observed across our panel of G12C/Di-R and RASi-R lines suggested a remarkably consistent phenotype that we hypothesized may enhance nab-paclitaxel delivery and sensitivity even under conditions of RAS-inhibitor resistance.

To test this, we first assessed the uptake of FITC-labeled BSA (FITC-BSA) in a panel of G12Di-R, RASi-R, and parental cell lines. Fluorescence microscopy imaging of internalized FITC-BSA demonstrated that albumin uptake was greater in both G12Di-R and RASi-R compared with parental cells (Fig. 4B). Flow cytometry analysis also indicated that G12Di-R and RASi-R lines internalized significantly higher amounts of FITC-BSA than their respective parental lines (Fig. 4C). These results suggest that RAS inhibitor-resistant lines utilize macropinocytosis not only to internalize polysaccharides, as supported by our results with fluorescent dextrans (Fig. 3C–F), but also to increase the uptake of proteins such as albumin.

We next performed colony formation assays to test the sensitivity of G12Di-R, RASi-R, and parental cell lines to nab-paclitaxel versus free paclitaxel (Fig. 4D; Supplementary Fig. S5A and S5B) and generated dose–response curves from relative colony growth (Fig. 4E). Comparison of GI_50_ values revealed that the growth of G12Di-R and RASi-R lines was significantly more sensitive to nab-paclitaxel than that of the parental cells. Conversely the growth of the parental lines was significantly more sensitive to free paclitaxel compared with that of all but one of the G12Di-R lines. PANC-1 G12Di-R and all RASi-R lines showed no significant difference in sensitivity to paclitaxel compared with parental lines (Fig. 4F).

Additionally, because we observed that the MEKi trametinib also upregulated macropinocytosis, we tested whether it also enhanced nab-paclitaxel sensitivity. First, we confirmed that MEKi induced greater uptake of albumin than DMSO control (Supplementary Fig. S6A and S6B). As an orthogonal approach, we treated the cells with increasing doses of MEKi for 24 to 120 hours and then incubated them in human serum albumin (HSA) for 1 hour prior to lysis. Immunoblotting for HSA confirmed that MEKi increased albumin internalization in a dose- and time-dependent manner (Supplementary Fig. S6C). Using a time frame suitable for potentially enhanced sensitivity due to increased albumin internalization, we treated cells for 72 hours with MEKi or DMSO and then performed 5-day dose–response proliferation assays in the presence or absence of nab-paclitaxel or free paclitaxel. We found that MEKi treatment decreased the IC_50_ value for nab-paclitaxel, indicating increased sensitivity, but had no significant impact on the response to free paclitaxel (Supplementary Fig. S6D and S6E). We then performed longer-term colony formation assays in cells treated for 72 hours with DMSO or MEKi followed by nab-paclitaxel (Supplementary Fig. S6F). We found that MEKi and nab-paclitaxel showed a synergistic effect on cell growth, as indicated by Bliss independence analysis of single-agent effect size (Supplementary Fig. S6G; ref. 44).

In summary, the increase in macropinocytosis resulting from treatment with G12Di, RASi, or MEKi both enhanced the uptake of albumin alone and sensitized cell growth to albumin-bound paclitaxel. These results suggest that inhibition of RAS or MEK induces a consistent and functional phenotype which has the potential to be exploited to enhance the uptake and efficacy of albumin-bound therapies.

### PDAC cell lines utilize both PI3K-dependent and -independent signaling pathways to support macropinocytosis

**Figure 5.**
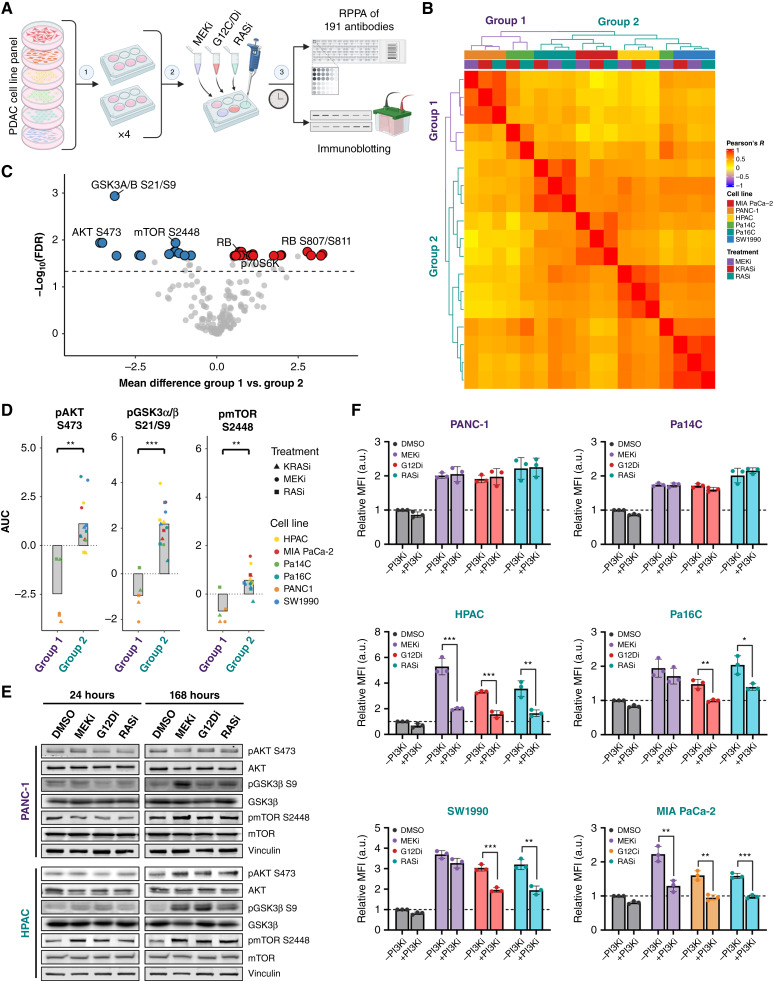
PDAC cell lines display both PI3K-dependent and -independent signaling supporting macropinocytosis. **A,** Schematic diagram delineating the experimental set-up for RPPA analysis and parallel validation via immunoblotting. **B,** RPPA Pearson correlation coefficients comparing cell lines and treatments based on the AUC following dimensionality reduction. Statistical analysis was performed from four biological replicates. Unsupervised clustering revealed group 1 (purple) and group 2 (teal) as designated on dendrogram. **C,** Differentially expressed (phospho)proteins between groups 1 and 2 following differential expression and *t* test comparisons. Data points indicate proteins or phospho-proteins with significantly lower levels (blue) or higher levels (red) in group 1 compared with group 2. **D,** Bar graphs of RPPA AUC values for selected antibodies comparing all cell lines and conditions from group 1 and group 2. **, ρ < 0.01; ***, ρ < 0.001, by standard paired *t* test with Benjamini–Hochberg adjustment for multiple comparisons. **E,** Immunoblotting of indicated proteins in PANC-1 and HPAC cells treated identically to RPPA samples with DMSO, MEKi, G12Di, or RASi for 24 or 168 hours. **F,** Macropinocytosis was measured via flow cytometry in *KRAS*-mutant PDAC cell lines that were treated with DMSO, MEKi, G12Di, or RASi for 168 hours with or without pictilisib (PI3Ki: 1 μmol/L for MIA PaCa-2, Pa16C, and HPAC; 3.3 μmol/L for PANC-1, Pa14C, and SW1990) for 24 hours prior to collecting cells for analysis. Macropinocytosis was quantified via TMR–dextran labeling. Data are presented as the mean ± SEM of three independent experiments. *, ρ < 0.05; **, ρ < 0.01, and ***, ρ < 0.001, by the unpaired Student *t* test, comparing presence or absence of PI3Ki within each group. MFI, median fluorescence intensity. [**A,** Created in BioRender. Robb, R. (2026) https://BioRender.com/44b6jcg.]

We next sought to determine the signaling dynamics associated with sustained RAS-inhibitor and MEK-inhibitor treatment that could support macropinocytosis. We performed RPPA pathway mapping to gain a comprehensive view of well-established signaling networks and how they change upon inhibition of KRAS, RAS, or MEK. We treated a panel of six PDAC cell lines with RASi, G12C/Di, or MEKi over a time course that included 24, 72, 120, and 168 hours (Fig. 5A). At early time points, we observed robust target inhibition, as indicated by decreased pERK (T202/Y204), and total MYC (Supplementary Figs. S7A and S8A). As reported previously (39, 45, 46), rebound in these canonical markers of RAS pathway signaling was observed at later time points to varying degrees across the cell line panel.

Across all time points, we observed increased levels of cyclin-dependent kinase inhibitor 1B (CDKN1B; p27; Supplementary Figs. S7A and S8A), an inhibitor of cyclin-dependent kinases, and of cyclin A2 (CCNA2; Supplementary Fig. S7A), consistent with cell-cycle arrest as a well-validated consequence of RAS pathway inhibition (47). Consistent with our observation of increased macropinocytosis following treatment with G12C/Di, RASi or MEKi, we also observed high levels of LAMP2 (Supplementary Figs. S7A and S8A), a major component of late endosomal and lysosomal membranes and a key facilitator of the fusion of macropinosomes with lysosomes for degradation (48, 49).

We observed considerable heterogeneity of signaling pathway activities between cell lines across time points, motivating us to perform more global analyses of our complex dataset. We first performed dimensionality reduction by calculating the area under the curve (AUC) across all time points for each protein or phosphoprotein, treatment, and cell line to represent signaling dynamics (Supplementary Fig. S8B). We then used the AUC values to perform PCA and unsupervised clustering of Pearson’s correlations of cell lines and treatments. We found that heterogeneity was driven primarily by cell lines and secondarily by treatments (Supplementary Fig. S8C). Unsupervised clustering of Pearson’s correlations led to separation of two groups, groups 1 and 2 (Fig. 5B). Group 1 consisted of all treatment conditions in PANC-1 cells as well as treatments with RASi and G12Di of Pa14C cells. Comparisons between these groups revealed several differentially regulated proteins and phosphoproteins (Fig. 5C). In particular, we observed a significant upregulation of phosphoproteins associated with PI3K signaling (i.e., pAKT S473, pGSK3α/β S29/S9, and pmTOR S2448) in group 2 but not group 1 (Fig. 5C–E; Supplementary Fig. S8D and S8E).

PI3K is a canonical driver of macropinocytosis (50, 51). Therefore, we next sought to determine whether PI3K signaling was required to drive the increase in macropinocytosis observed following direct RAS-inhibitor and MEK-inhibitor treatment. We tested whether inhibition of PI3K/AKT signaling with the pan-PI3K inhibitor pictilisib (PI3Ki) could blunt G12C/Di-, RASi- and MEKi-induced macropinocytosis. We first determined the doses at which maximal target inhibition was achieved and assessed growth inhibition at those doses (Supplementary Fig. S9A and S9B). Next, we measured macropinocytosis in G12C/Di-, RASi-, or MEKi-treated cells with or without PI3Ki and found that PI3Ki blunted RAS pathway inhibitor–induced macropinocytosis in group 2 but not group 1 cell lines (Fig. 5F). We conclude that upregulation of PI3K–AKT signaling in response to RAS pathway inhibition is a marker of PI3K-dependent macropinocytosis and that not all PDAC cell lines depend on PI3K signaling to upregulate macropinocytosis in response to RAS or MEK inhibition.

### RAS inhibitor-resistant PDAC cell lines depend on distinct pathways that converge on activation of RAC1 and drive macropinocytosis

**Figure 6.**
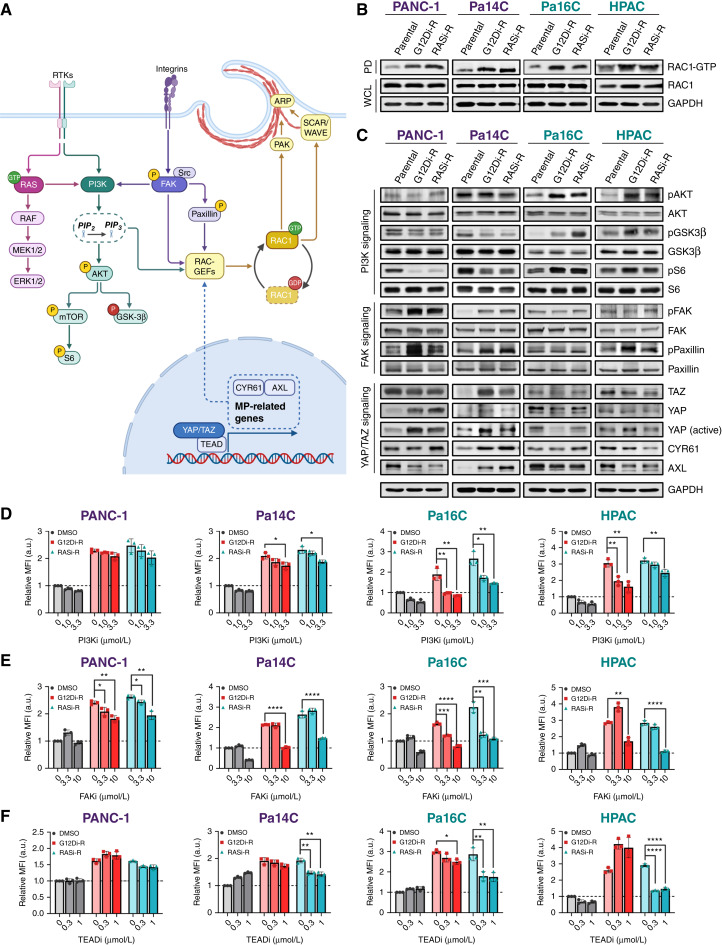
Different RAS inhibitor-resistant PDAC cell lines activate RAC via distinct pathways to upregulate macropinocytosis. **A,** Diagram of signaling pathways relevant to macropinocytosis that focuses on the central role of RAC activation downstream of RAS, PI3K, and FAK signaling. **B,** Immunoblotting of indicated parental, MRTX1133- (G12Di-R), or RMC7797-resistant (RASi-R) KRAS-mutant PDAC cell lines from RAC1-GTP pulldown (PD) assay showing active RAC1-GTP levels and indicated proteins from corresponding whole-cell lysates (WCL). **C,** Immunoblots of indicated parental, MRTX1133- (G12Di-R), or RMC7977-resistant (RASi-R) KRAS-mutant PDAC cell lines. GAPDH levels were used to monitor equivalent total protein loading. Blots are representative of three independent experiments. **D** and **E,** Macropinocytosis was measured via flow cytometry in indicated parental, MRTX1133- (G12Di-R), or RMC7977-resistant (RASi-R) KRAS-mutant PDAC cell lines with or without indicated doses of pictilisib (PI3Ki; **D**), defactinib (FAKi; **E**), and IAG-933 (**F**). Macropinocytosis was quantified via TMR–dextran labeling. Data are presented as the mean ± SEM of three independent experiments. *, ρ < 0.05, and **, ρ < 0.01, by the unpaired Student *t* test, comparing each PI3Ki dose against DMSO for each line. *, ρ < 0.05; **, ρ < 0.01; ***, ρ < 0.001, and ****, ρ < 0.0001, by the unpaired Student *t* test, comparing each inhibitor dose against DMSO for each line. MFI, median fluorescence intensity; MP, macropinocytic; pPaxillin, phospho-paxillin. [**A,** Created in BioRender. Robb, R. (2026) https://BioRender.com/iv8tuuh.]

Activation of the small GTPase, RAC1, plays a critical role in controlling membrane ruffling and macropinosome formation (52, 53), following the activation of several distinct upstream signaling pathways, including PI3K, FAK, and YAP1 and WWTR1, commonly referred to as YAP and TAZ (YAP/TAZ; refs. 54, 55), as depicted in Fig. 6A. Accordingly, we hypothesized that the enhanced macropinocytosis we observed in G12Di-R and RASi-R lines compared with parental lines may be associated with higher basal levels of active RAC1. To test this, we performed RAC1 activation pulldown assays to assess the levels of RAC-GTP. Immunoblot analysis of the RAC1-GTP pulldown samples showed higher levels of active RAC1 in G12Di-R and RASi-R lines when compared with matched parental lines (Fig. 6B). Densitometry quantitation of RAC1-GTP normalized to total RAC1 from parallel whole-cell lysates revealed a 2- to 4-fold increase in relative RAC1 activation (Supplementary Fig. S10A).

PI3K and integrin–FAK signaling are critical regulators of macropinocytosis in multiple solid tumor types (31, 34, 40). Upregulated PI3K and FAK signaling have each been shown to result in increased activation of RAC1 (50, 51, 56) as well as resistance to RAS inhibition (24, 57, 58). To assess whether PI3K and/or FAK signaling were also upregulated in G12Di-R and RASi-R PDAC cell lines, we performed immunoblot analysis of key phosphorylated and total proteins in each pathway (Fig. 6C). Consistent with our RPPA results, G12Di-R and RASi-R group 2 cell lines showed higher activation of PI3K pathway–associated proteins, such as pAKT S473, pGSK3B S9, and pS6 S235/S236, whereas G12Di-R and RASi-R group 1 cell lines showed an apparent downregulation (Fig. 6C; Supplementary Fig. S10B and S10C). In contrast, activation of FAK (phospho-FAK Y397) and its direct target paxillin (phospho-paxillin Y118) was strongly upregulated in G12Di-R and RASi-R group 1 cell lines (Fig. 6C; Supplementary Fig. S10D and S10E). Therefore, we hypothesized that different upstream activators lead to macropinocytosis in these two groups and that macropinocytosis would be sensitive to PI3Ki in group 2 cell lines and to FAKi (defactinib) in group 1 cell lines.

To test this, we first performed Western blot analysis of our parental, G12Di-R, and RASi-R cell lines treated with increasing doses of either PI3Ki (Supplementary Fig. S11A) or FAKi (Supplementary Fig. S12A) to determine the doses at which maximal target inhibition was achieved. We next assessed macropinocytic uptake of TMR-Dex in parental, G12Di-R, and RASi-R cell lines treated with increasing doses of either PI3Ki or FAKi for 24 hours. We found that PI3Ki treatment did indeed cause a marked reduction in macropinocytosis in resistant cell lines in group 2 (Fig. 6D). The highest dose of PI3Ki tested also led to a small but statistically significant reduction in macropinocytosis in G12Di-R and RASi-R Pa14C cells (group 1; Fig. 6D), indicating that signaling does not always directly correlate with macropinocytic dependency. Strikingly, although PI3Ki treatment reduced PI3K activation to basal levels if not further (Supplementary Fig. S11A), it did not always reduce macropinocytosis to the levels observed in parental cell lines (Fig. 6D), indicating that other pathways contribute to macropinocytosis even in those lines that are PI3Ki-sensitive. This observation further supports our conclusion that multiple signaling pathways mediate macropinocytosis in the context of RAS-inhibitor resistance.

Unexpectedly, when we treated the panel of parental and RAS inhibitor-resistant models with FAKi, we observed reduced macropinocytic uptake in all G12Di-R and RASi-R lines, with most decreasing to levels similar to those seen in the respective parental lines (Fig. 6E). We speculated that this could be due to FAKi-related suppression of PI3K signaling (Supplementary Fig. S12A), as defactinib causes PI3K to dissociate from FAK (59, 60) and blocks integrin-mediated signaling to PI3K (Fig. 6A; refs. 59, 60). In agreement with this, FAKi treatment did lead to reduced PI3K signaling in all cell lines tested (Supplementary Fig. S12A). Furthermore, combined inhibition of PI3K and FAK reduced macropinocytic uptake to a greater extent than single-agent treatment in a subset of resistant lines (Supplementary Fig. S12B). Together, these findings suggest that PI3K- and FAK-dependent signaling can support macropinocytosis through both overlapping as well as partially nonredundant mechanisms, with the relative contribution of each pathway varying across RAS inhibitor-resistant models.

Because neither PI3K nor FAK inhibition uniformly suppressed macropinocytosis across all resistant models, we next asked whether additional resistance-associated signaling programs could contribute to increased macropinocytosis. YAP and TAZ are transcriptional coactivators that signal through TEAD transcription factors and have been implicated in both resistance to RAS inhibition and regulation of macropinocytosis (Fig. 6A; refs. 61, 62). Furthermore, immunoblotting for key YAP/TAZ proteins revealed that multiple resistant lines exhibit significant differences in expression of YAP, TAZ, and/or downstream transcriptional targets compared with the respective parental lines, with robust upregulation observed in some resistant lines and downregulation in others (Fig. 6C; Supplementary Fig. S13A and S13B). Therefore, we tested whether TEAD inhibition could reduce macropinocytic uptake in our G12Di-R and RASi-R lines. TEAD inhibition reduced macropinocytosis in a subset of resistant lines (Fig. 6F; Supplementary Fig. S13C), indicating that YAP/TAZ–TEAD-dependent transcriptional programs may contribute to this phenotype in select resistant states. Although we observed increased expression YAP/TAZ-associated proteins in some resistant lines, these markers did not directly correlate with TEAD inhibitor sensitivity with respect to impact on macropinocytosis, suggesting that YAP/TAZ pathway expression alone does not predict TEAD-dependent macropinocytic regulation.

Together, these findings extend our PI3K and FAK inhibitor results and support a model in which RAS inhibitor-resistant PDAC cells sustain elevated macropinocytosis through heterogeneous, partially overlapping signaling dependencies. We conclude that the upregulated macropinocytosis associated with RAS-inhibitor resistance is a convergent phenotype mediated by multiple signaling pathways, including PI3K-, FAK-, and TEAD-associated mechanisms.

## Discussion

Despite recent progress in pharmacologically targeting RAS, durable responses to direct RAS inhibitors remain rare (19), highlighting the remarkable adaptability of *RAS*-mutant tumors. Our findings revealed that macropinocytosis, a hallmark nutrient scavenging process attributed to oncogenic RAS activity (29–31), is not simply lost upon RAS pathway inhibition in *KRAS*-mutant PDAC cell lines. Rather, macropinocytic activity rebounded and was subsequently enhanced following prolonged RAS suppression. This adaptive upregulation occurred across genetic and pharmacologic inhibition of RAS, indicating that *KRAS*-mutant PDAC cells possess intrinsic signaling flexibility that enables them to maintain macropinocytic capacity despite sustained oncogenic blockade. Furthermore, we demonstrated that enhanced macropinocytic uptake was maintained in RAS inhibitor-resitant cell lines. Upregulation of macropinocytosis was observed in both human- and KPC-derived cell lines generated both *in vitro* and *in vivo*, respectively. Proteomics revealed that expression of macropinocytosis-related proteins increased over time following RASi treatment. We also identified PI3K-, FAK-, and TEAD-associated signaling dependencies that support macropinocytic uptake in subsets of RAS inhibitor-resistant cells, revealing that this process can be enhanced through multiple compensatory routes that likely converge on cytoskeletal remodeling and RAC1-dependent macropinosome formation. Importantly, we also demonstrated that resistant cell lines with elevated macropinocytosis exhibited enhanced uptake of albumin and greater sensitivity to albumin-bound chemotherapy. These results position enhanced macropinocytosis as an adaptive, resistance-linked phenotype that persists under therapeutic pressure and highlight a potential opportunity to exploit this process for improved drug delivery strategies in the RAS-inhibited state.

Because macropinocytosis can be triggered by diverse cellular stresses, including nutrient deprivation, oxidative stress, and drug exposure (31), we hypothesized that acute RAS inhibition might initially elevate macropinocytosis as a stress response but that this would normalize once cells adapted to chronic treatment. Contrary to this expectation, RAS inhibitor-resistant cell lines maintained or further increased macropinocytic uptake compared with their parental counterparts. This durable phenotype suggests that sustained pathway suppression selects for signaling adaptations or transcriptional programs that preserve high macropinocytic capacity. In agreement with this, emerging evidence suggests that macropinocytosis can be enhanced and can contribute to drug resistance to mutant-selective KRAS inhibitors (63, 64). MIA PaCa-2 cells with acquired resistance to the KRAS^G12C^-selective inhibitor, sotorasib (63), and PANC-1 cells derived from xenograft tumors refractory to treatment with the KRAS^G12D-selective^ inhibitor MRTX1133 (64), exhibit higher levels of macropinocytosis than vehicle-treated cells. Notably, both studies also implicate RAC1-dependent mechanisms in this adaptive phenotype. Li and colleagues (64) showed that MRTX1133-refractory PDAC cells engage an AGER–DIAPH1–RAC1 signaling axis to drive macropinocytosis, whereas Theardy and colleagues (63) found that KRAS^G12C^ inhibitor–resistant cells restore macropinocytosis through a YAP1–SDC1 pathway associated with increased RAC1 activity. Together with our finding that RAS inhibitor-resistant PDAC cells exhibit increased RAC1 activation, these studies support a model in which heterogeneous resistance-associated pathways converge on RAC1-dependent macropinocytic remodeling following RAS pathway inhibition.

Mechanistically, multiple interconnected signaling pathways implicated in resistance to RAS-inhibitor treatment are also known to stimulate macropinocytosis, suggesting that acquisition of RAS-inhibitor resistance and upregulation macropinocytosis are mechanistically intertwined. These include increased signaling from receptor tyrosine kinases (RTK; refs. 19, 20, 24, 65–67), PI3K-AKT (19, 20, 24, 29, 30), FAK (58), and WNT/GSK3 (68–71) pathways as well as transcriptional regulation by YAP/TAZ (61, 62), nuclear factor erythroid 2–related factor 2 (NFE2L2; NRF2; refs. 3, 72), and catenin beta 1 (CTNNB1; β-catenin; refs. 68, 71, 73). Herein, we investigated the contributions of PI3K–AKT, FAK, and YAP/TAZ–TEAD signaling to increased macropinocytosis in RAS inhibitor-resistant cell lines. PI3K and FAK contribute to an interconnected signaling network that converges on RAC1 to regulate macropinocytosis (29, 74). Each pathway promotes RAC1 activation through distinct GEFs: ELMO/DOCK180 and GIT/PIX downstream of FAK (75–77), TIAM1 downstream of PI3K (78), as well as the shared RAC-GEF, VAV2 (79). Consistent with this, we observed that RAS inhibitor-resistant cells exhibited both increased activation of RAC1 and enhanced macropinocytosis which was blunted by inhibition of PI3K and/or FAK to varying degrees across cell lines. Additionally, we found that in PI3Ki-responsive lines, FAK inhibition also reduced macropinocytic uptake, suggesting cooperation between these pathways. This is in line with previous work which has shown that FAK activates PI3K, amplifying its signaling (74).

Transcriptional reprogramming mediated by YAP/TAZ has also been implicated in both RAS-inhibitor resistance as well as macropinocytosis regulation (61, 62). YAP/TAZ activation has been shown to drive resistance to RAS pathway inhibitors by restoring proliferative and survival signaling through transcriptional upregulation of growth-promoting and antiapoptotic genes (62). In parallel, YAP/TAZ enhances macropinocytic uptake by inducing expression of regulators of cytoskeletal and membrane dynamics (61), thereby reinforcing nutrient scavenging and metabolic adaptation under sustained RAS inhibition. Consistent with this, we found that TEAD inhibition reduced macropinocytosis in a subset of RAS inhibitor-resistant lines, indicating that YAP/TAZ–TEAD-dependent transcriptional programs can also contribute to enhanced macropinocytic uptake in select resistant states. However, changes in YAP, TAZ, or downstream target expression did not directly predict whether TEAD inhibition would suppress macropinocytosis. This suggests that TEAD-dependent macropinocytic regulation may be influenced by additional factors beyond steady-state expression of individual YAP/TAZ pathway markers, including YAP/TAZ localization, TEAD transcriptional output, cell-state context, or compensation by parallel signaling pathways. Notably, none of the pathway inhibitors tested uniformly blocked macropinocytosis across all resistant models, suggesting that multiple compensatory mechanisms can contribute to the induction of macropinocytic activity.

In addition to these signaling adaptations, our proteomic analysis revealed coordinated upregulation of core macropinocytic machinery, including proteins involved in membrane ruffling, cargo uptake, vesicle maturation, and degradation. These findings suggest that increased macropinocytosis in the resistant state is reinforced not only by signaling through PI3K-, FAK-, and TEAD-associated signaling but also by a broader remodeling of the endocytic and cytoskeletal proteome that enhances the cell’s capacity for macropinocytic uptake.

Beyond PI3K, FAK, and YAP/TAZ-TEAD signaling, other related pathways could be contributing to the increase in macropinocytosis in *KRAS*-mutant PDAC upon RAS-inhibitor treatment. Importantly, recurrent mechanisms of resistance to RAS-inhibitor treatment include the upregulation of signaling pathways upstream of RAS, allowing reactivation of PI3K–AKT and RAS–MAPK signaling (19, 20, 24, 80). For instance, multiple studies have found that RAS inhibitor-resistant cells harbor activating mutations or amplifications of RTKs (19, 20, 24, 80). RTKs lie upstream of RAS–MAPK, PI3K–AKT (81), and FAK–SRC (74) signaling pathways, all of which influence RAC1 activity and cytoskeletal remodeling required for membrane ruffling and vesicle formation (29, 50, 51, 56, 74). Other transcriptional or stress-adaptive regulators, including NRF2 and β-catenin, may also contribute to the broader rewiring of macropinocytic capacity in resistant cells. The overlap between these pathways and others highlights the extensive cross-talk between RAS-inhibitor resistance and macropinocytic regulation, indicating that there is unlikely to be a single dominant driver of macropinocytosis in resistant cells. Although defining the baseline cellular or genetic features that predispose individual PDAC cell lines to develop PI3K-, FAK-, or TEAD-associated macropinocytic dependencies was beyond the scope of this study, such determinants are likely to be important for understanding the trajectory of adaptation to RAS inhibition. Future studies integrating baseline RTK expression, mutational status, and pathway activity with acquired resistance phenotypes may help identify predictive biomarkers that guide rational combination strategies to prevent or delay resistance.

Likewise, due to the extensive number of adaptations which contribute to enhanced macropinocytosis following RAS pathway inhibition, it could prove difficult to find a consistent way to inhibit this enhancement. Therefore, because upregulated macropinocytosis was such a consistent phenotype across RAS inhibitor-resistant PDAC cells, we propose that exploiting macropinocytosis for enhanced tumor-selective drug delivery might provide a more reliable approach for the design of RAS inhibitor-based drug combinations (40). Previous work has shown that elevated macropinocytosis in tumor cells can be therapeutically leveraged for selective uptake of macromolecular drugs and nanoparticles (40, 41). We found that RAS inhibitor-resistant cells exhibit greater uptake of albumin and enhanced sensitivity to nab-paclitaxel, an albumin-bound chemotherapeutic used as a standard of care in PDAC (43). Moreover, pharmacologic induction of macropinocytosis using a MEKi increased nab-paclitaxel sensitivity in drug-naïve cells, highlighting a potentially exploitable synergy between RAS pathway inhibition and albumin-bound therapeutics. Consistent with these findings, a recent study (24) demonstrated that combining a mutation-selective inhibitor of KRAS^G12D^ (MRTX1133) with gemcitabine and nab-paclitaxel led to markedly improved tumor control and prolonged survival in the *Kras*^*G12D*^-mutant KPC mouse model of PDAC compared with either monotherapy or dual combinations. This further supports the potential benefit of integrating KRAS inhibition with standard chemotherapy. Because macropinocytosis mediates the internalization of diverse macromolecular carriers, this phenotype could also potentiate uptake of other nanoparticle-based or antibody–drug conjugate therapies (40, 41). For example, nab-sirolimus (an albumin-bound mTOR inhibitor; ref. 82) represents an attractive candidate. Previous studies have demonstrated that combining RAS inhibitors or MEK inhibitors with inhibitors of mTOR causes synergistic inhibition of cell growth and induction of apoptosis *in vitro* and *in vivo* (83, 84). RAS pathway inhibition may result in greater uptake of nab-sirolimus, thereby further improving its antitumoral efficacy.

Together, our findings highlight macropinocytosis as a plastic and targetable metabolic process that may emerge as a resistance-associated adaptation in *KRAS*-mutant PDAC. Elevated macropinocytosis may persist or even intensify in response to direct RAS inhibition, offering a window for rational combination strategies aimed at improving treatment responses.

## Supplementary Material

Supplementary Table S1 Antibodies used for RPPA analysis.

Figure S1 KRAS suppression results in a transient reduction in macropinocytosis

Figure S2 Prolonged RAS pathway inhibition in KRAS-mutant PDAC cells results in increased macropinocytosis

Figure S3 Cell lines with acquired resistance to RAS inhibitors exhibit a flattened cellular morphology

Figure S4 Changes to the total proteome following RASi treatment and upon the acquisition of RASi resistance

Figure S5 RAS inhibitor treatment enhances the sensitivity of PDAC cell lines to albumin-bound nab-paclitaxel but not free paclitaxel

Figure S6 MEK inhibitor treatment enhances the sensitivity of PDAC cell lines to albumin-bound nab-paclitaxel but not free paclitaxel

Figure S7 RPPA analysis of PDAC cell line panel treated with RAS ERK MAPK inhibitors for 24 to 168 hours

Figure S8 PDAC cell lines display both PI3K-dependent and -independent signaling supporting macropinocytosis

Figure S9 Pictilisib treatment impairs growth and PI3K signaling in PDAC cell lines

Figure S10 Activation of the PI3K and FAK pathways results in increased RAC activity in RASi-resistant cell lines

Figure S11 Pictilisib (PI3Ki) treatment decreases PI3K-mediated signaling in PDAC cell lines

Figure S12 Defactinib (FAKi) treatment decreases FAK-mediated signaling and macropinocytosis in PDAC cell lines

Figure S13 A subset of RASi-resistant PDAC cell lines activates YAP and/or TAZ to upregulate macropinocytosis

## Data Availability

All data needed to evaluate the conclusions in the article are present in the main text or the supplementary materials or have been uploaded to public repositories (below). The mass spectrometry proteomics data have been deposited to the ProteomeXchange Consortium via the PRIDE (85) partner repository with the dataset identifier PXD066616 (https://www.ebi.ac.uk/pride/archive/projects/PXD066616). Any other data associated with the study will be provided by K.L. Bryant upon request.
