# Supplementary material for: Acquisition of Resistance to RAS Inhibition Is Associated with the Upregulation of Macropinocytosis through Both PI3K-Dependent and -Independent Signaling
Source: Cancer Res Commun. 2026 Jul 28;6(7):1794–813. doi: 10.1158/2767-9764.CRC-25-0731 (PMC13410306; doi:10.1158/2767-9764.CRC-25-0731)
Supplement: Supplementary Table S1 — Antibodies used for RPPA analysis. [file crc-25-0731_supplementary_table_s1_suppst1.docx]

**Supplementary Table 1.** Antibodies used for RPPA Analysis.

| Cat # | Antibody | Company | Dilution for Array | Host |
| --- | --- | --- | --- | --- |
| 7413 | 14-3-3 zeta, delta | CellSig | 1:100 |  |
| 9452 | 4E-BP1 | CellSig | 1:200 | R |
| 9451 | 4E-BP1 (S65) | CellSig | 1:50 | R |
| 9455 | 4E-BP1 (T70) | CellSig | 1:200 | R |
| 05-321 | 4G10 (anti Phosphotyrosine) | Upstate | 1:1000 | M |
| 9272 | Akt | CellSig | 1:2000 | R |
| 4060 | AKT (S473) XP | CellSig | 1:100 | R |
| 9275 | AKT (T308) | CellSig | 1:100 | R |
| 3348 | ALK (Y1586) | CellSig | 1:200 | RmAb |
| 3341 | ALK (Y1604) | CellSig | 1:50 | R |
| 4188 | AMPKalpha (T172) (D79.5E) | CellSig | 1:2000 | RmAb |
| 4184 | AMPKalpha1 (S485) | CellSig | 1:50 | R |
| 4431 | A-Raf (S299) | CellSig | 1:50 | R |
| 2010 | ATG12 | CellSig | 1:100 | R |
| 2630 | ATG5 | CellSig | 1:1000 | R |
| 5883 | ATM (S1981) (D6H9) | CellSig | 1:400 | RmAb |
| 2853 | ATR (S428) | CellSig | 0.0764 | R |
| 2914 | Aurora A (T288)/B (T232)/C (T198) (D13A11) | CellSig | 1:50 | RmAb |
| 3092 | Aurora A/AIK | CellSig | 1:50 | R |
| 2087 | Axin1 (C76H11) | CellSig | 1:500 |  |
| 4566 | Axl (C44G1) | CellSig | 1:200 | RmAb |
| 5724 | Axl (Y702) | CellSig | 1:50 | R |
| 9291 | BAD (S112) | CellSig | 1:200 | R |
| 9295 | BAD (S136) | CellSig | 1:50 | R |
| 9297 | BAD (S155) | CellSig | 1:100 | R |
| 3738 | Beclin 1 | CellSig | 1:100 | R |
| 2933 | BIM | CellSig | 1:500 | R |
| H00000645-M09 | BLVRB (biliverdin reductase B) (2F4) | Abnova | 1:50 | M |
| 2696 | B-Raf (S445) | CellSig | 1:50 | R |
| 7237 | Caspase 9, cleaved (D330) (D2D4) | CellSig | 1:200 | RmAb |
| 9661 | Caspase-3, cleaved (D175) | CellSig | 1:50 | R |
| 9761 | Caspase-6, cleaved (D162) | CellSig | 1:50 | R |
| 9491 | Caspase-7, cleaved (D198) | CellSig | 1:100 | R |
| 9505 | Caspase-9, cleaved (D315) | CellSig | 1:100 | R |
| 9562 | Catenin (beta) | CellSig | 1:100 | R |
| 9561 | Catenin (beta) (S33/37/T41) | CellSig | 1:100 | R |
| 3373 | Cathepsin B (G60) | CellSig | 1:500 | R |
| 2267-1 | Caveolin-1 (Y14) (EPR2288Y) | Epitomics | 1:200 | RmAb |
| 16399 | CD28 (Y191) [E5B9Z] | CellSig | 1:100 | R |
| 4539 | cdc2 (Y15) (10A11) | CellSig | 1:50 | RmAb |
| SAB4300114 | CDC25A (S76) | Sigma | 1:100 | R |
| 2546 | CDK2 (78B2) | CellSig | 1:200 | R |
| 12790 | CDK4 (D9G3E) | CellSig | 1:200 | RmAb |
| 13331 | CDK6 (D4S8S) | CellSig | 1:50 | RmAb |
| 2341 | Chk-1 (S345) | CellSig | 1:50 | R |
| 2665 | Chk-2 (S33/35) | CellSig | 1:50 | R |
| 3073 | c-Kit (Y703) (D12E12) | CellSig | 1:50 | RmAb |
| 9402 | c-Myc | CellSig | 1:100 | R |
| 13748 | c-Myc (S62) (E1J4K) | CellSig | 1:100 | RmAb |
| 3318 | Cofilin (D59) | CellSig | 1:100 | R |
| 3313 | Cofilin (S3) (77G2) | CellSig | 1:500 | RmAb |
| 2831 | c-PLA2 (S505) | CellSig | 1:1000 | R |
| 9427 | c-Raf (S338) (56A6) | CellSig | 1:200 | RmAb |
| 9196 | CREB (S133) (1B6) | CellSig | 1:200 | M |
| 4656 | Cyclin A2 (BF683) | CellSig | 1:50 | M |
| 4135 | Cyclin B1 (V152) | CellSig | 1:200 | M |
| 2978 | Cyclin D1 (92G2) | CellSig | 1:100 | RmAb |
| 2936 | Cyclin D3 (DCS22) | CellSig | 1:200 | M |
| 20808 | Cyclin E1 (D7T3U) | CellSig | 1:100 | RmAb |
| 4132 | Cyclin E2 | CellSig | 1:200 | R |
| 12574 | DAG Lipase Beta | CellSig | 1:50 |  |
| 06-463 | DEPTOR | Millipore | 1:4000 | R |
| 11-547-1-AP | DGK | ProteinTech | 1:250 | R |
| 4687 | DKK1 | CellSig | 1:200 | R |
| 3224 | Dvl2 (30D2) | CellSig | 1:1000 |  |
| 3218 | Dvl3 | CellSig | 1:1000 |  |
| 2238 | EGFR (S1046/1047) | CellSig | 1:500 | R |
| 2237 | EGFR (Y1045) | CellSig | 1:50 | R |
| 2234 | EGFR (Y1068) | CellSig | 1:50 | R |
| ab76195 | EGFR (Y1101) (M199) | Abcam | 0.0764 | M |
| 44-792G | EGFR (Y1148) | ThermoFish | 1:100 | R |
| 44-794G | EGFR (Y1173) | ThermoFish | 1:100 | R |
| 2231 | EGFR (Y845) | CellSig | BAD Ab | R |
| 2235 | EGFR (Y992) | CellSig | 1:50 | R |
| 9741 | eIF4E (S209) | CellSig | 1:50 | R |
| 2498 | eIF4G | CellSig | 1:200 | R |
| 2441 | eIF4G (S1108) | CellSig | 1:1000 | R |
| 9181 | Elk-1 (S383) | CellSig | 1:100 | R |
| 9102 | ERK 1/2 | CellSig | 1:200 | R |
| 9101 | ERK 1/2 (T202/Y204) | CellSig | 1:1000 | R |
| 3141 | Ezrin (T567)/Radixin (T564)/Moesin (T558) | CellSig | 1:100 | R |
| 3144 | Ezrin (Y353) | CellSig | 1:100 | R |
| 611806 | FAK (Y397) (18) | BD | 1:50 | M |
| 3281 | FAK (Y576/577) | CellSig | 1:200 | R |
| 3231 | Gab1 (Y627) | CellSig | 1:1000 | R |
| PA5-46830 | GABRP |  |  |  |
| 87985 | Galectin-3/LGALS3 (D412R) | CellSig | 1:300 |  |
| 5174 | GAPDH (D16H11) | CellSig | 1:100 | RmAb |
| 3972 | GRB2 | CellSig | 1:1000 | R |
| 9331 | GSK-3a/B (S21/9) | CellSig | 1:100 | R |
| 9337 | GSK-3alpha (S21) (46H12) | CellSig | 1:100 | M |
| 9315 | GSK-3beta | CellSig | 1:100 | R |
| SPA-894 | Heme-Oxygenase-1 | Stressgen | 1:500 | R |
| 610958 | HIF-1alpha (54) | BD | 1:50 | M |
| 9718 | Histone H2A.X (S139) | CellSig | 1:50 | R |
| 06-570 | Histone H3 (S10) Mitosis Marker | Upstate | 1:200 | R |
| 9753 | Histone H3, Di-Methyl (Lys9) | CellSig | 1:500 | R |
| ab9572 | IGF1 | Abcam | 1:250 | R |
| 3021 | IGF-1 Rec (Y1131)/Insulin Rec (Y1146) | CellSig | 1:500 | R |
| 3027 | IGF-1 Receptor beta | CellSig | 1:1000 | R |
| 3024 | IGF-1R (Y1135/36)/IR (Y1150/51) (19H7) | CellSig | 1:500 | RmAb |
| 2386 | IRS-1 (S612) | CellSig | 1:200 | R |
| sc-18822 | LAMP-2 (H4HB4) | SantaCruz | 1:2000 | M |
| 2775 | LC3B | CellSig | 1:100 | R |
| 3055 | LKB1 (S334) | CellSig | 1:50 | R |
| 2560 | LRP6 (C5C7) | CellSig | 1:200 | RmAb |
| 3155 | M-CSF Receptor (Y723) (49C10) | CellSig | 1:100 | RmAb |
| 9128 | MEK1 (S298) | CellSig | 1:2000 | R |
| 9122 | MEK1/2 | CellSig | 1:500 | R |
| 9121 | MEK1/2 (S217/221) | CellSig | 1:200 | R |
| 12888 | Merlin (D3S3W) | CellSig | 1:200 |  |
| 13281 | Merlin (S518) (D5A4I) | CellSig | 1:250 | RmAb |
| 3126 | Met (Y1234/1235) | CellSig | 1:200 | R |
| 9482 | Mitofusin-2 (D2D10) | CellSig | 1:500 |  |
| SOD-110 | Mn Superoxide Dismutase (SOD) | AssayDesign | 1:500 | R |
| 2972 | mTOR | CellSig | 1:200 | R |
| 2971 | mTOR (S2448) | CellSig | 1:100 | R |
| 3033 | NF-kappaB p65 (S536) (93H1) | CellSig | 1:200 | RmAb |
| sc-32793 | NQO1 | SantaCruz | 1:200 | M |
| ab62352 | Nrf2 (EP1808Y) | Abcam | 1:200 | R |
| MABE1328 | p16 INK4a (13H4.1) | Millipore | 1:50 | M |
| 2946 | p21 Waf1/Cip1 (DCS60) | CellSig | 1:200 | M |
| 610242 | p27 Kip1 | BD | 1:100 | M |
| 9211 | p38 MAPK (T180/Y182) | CellSig | 1:100 | R |
| 9282 | p53 | CellSig | 1:5000 | R |
| 9284 | p53 (S15) | CellSig | 1:1000 | R |
| 8025 | p62/SQSTM1 (D5E2) | CellSig | 1:50 | RmAb |
| 9202 | p70 S6 Kinase | CellSig | 1:100 | R |
| 9205 | p70 S6 Kinase (T389) | CellSig | 1:100 | R |
| 9341 | p90RSK (S380) | CellSig | 1:200 | R |
| 9344 | p90RSK (T359/S363) | CellSig | 1:200 | R |
| 2605 | PAK1 (S199/204)/PAK2 (S192/197) | CellSig | 1:50 | R |
| 2601 | PAK1 (T423)/PAK2 (T402) | CellSig | 1:100 | R |
| 2608 | PAK2 |  |  |  |
| 2607 | PAK2 (S20) | CellSig | 1:100 | R |
| 9532 | PARP (46D11) | CellSig | 1:100 | RmAb |
| 9541 | PARP, cleaved (D214) | CellSig | 1:100 | R |
| 69363 | Paxillin (Y118) (E9U9F) | CellSig | 1:200 | RmAb |
| 3169 | PDGF Receptor beta (28E1) | CellSig | 1:200 | RmAb |
| 3161 | PDGF Receptor beta (Y751) | CellSig | 1:50 | R |
| 3061 | PDK1 (S241) | CellSig | 1:200 | R |
| 610045 | PI3-Kinase | BD | 1:100 | M |
| 4252 | PI3-Kinase p110gamma | CellSig | 1:100 | R |
| 4781 | PKA C (T197) | CellSig | 1:200 | R |
| 9371 | PKC (pan) (betaII S660) | CellSig | 1:200 | R |
| 2822 | PLC-gamma-1 | CellSig | 1:500 | R |
| 2821 | PLCgamma1 (Y783) | CellSig | 1:100 | R |
| 558400 | PLK1 (T210) | BD | 1:200 | M |
| 2581 | PP1a (T320) | CellSig | 1:100 | R |
| 2039 | PP2A a Subunit | CellSig | 1:1000 | R |
| 4953 | PP2A B Subunit | CellSig | 1:1000 | R |
| 44-1100 | PRAS40 (T246) | BioSource | 1:1000 | R |
| 4438 | PRMT4/CARM1 |  |  |  |
| 9552 | PTEN | CellSig | 1:50 | R |
| 9551 | PTEN (S380) | CellSig | 1:500 | R |
| 4976 | PUMA | CellSig | 1:200 | R |
| 8875 | RAD51 (D4B10) | CellSig | 1:250 | RmAb |
| 05-516 | Ras (RAS10) | Upstate | 1:200 | M |
| 3339 | Ras total | CellSig | 1:200 | R |
| 3322 | Ras-GRF1 | CellSig | 1:200 | R |
| 3321 | Ras-GRF1 (S916) | CellSig | 1:50 | R |
| 9309 | Rb (4H1) | CellSig | 1:500 | M |
| 3590 | Rb (S780) | CellSig | 1:2000 | R |
| 9308 | Rb (S807/811) | CellSig | 1:200 | R |
| 9348 | RSK3 (T356/S360) | CellSig | 1:500 | R |
| 4856 | S6 Ribosomal Protein (S235/236) (2F9) | CellSig | 1:200 | R |
| 07-315 | SGK1 | Upstate | 1:200 | R |
| 5599 | SGK1 (S78) (D36D11) | CellSig | 1:100 | R |
| 3941 | SHIP1 (Y1020) | CellSig | 1:50 | R |
| 5431 | SHP2 (Y580) (D66F10) | CellSig | 1:50 | RmAb |
| 4719 | Snail (SN9H2) | CellSig | 1:2000 | Rat |
| 2105 | Src (Y527) | CellSig | 1:200 | R |
| 2101 | Src Family (Y416) | CellSig | 1:100 | R |
| 2808 | Survivin (71G4) | CellSig | 1:500 | RmAb |
| 2711 | Syk (Y525/526) | CellSig | 1:50 | R |
| 36-2900 | Syndecan-1 (CD138) | Zymed | 1:200 | R |
| 3709 | TGF-Beta (56E4) | CellSig | 1:1000 | R |
| ab9635 | TNF alpha | Abcam | 1:200 | R |
| 13017 | TORC2/CRTC2 (5B10) |  |  | M |
| 3614 | Tuberin/TSC2 (Y1571) | CellSig | 1:50 | R |
| 3111 | VASP (S157) | CellSig | 1:100 | R |
| 4657 | VAV-1 | CellSig | 1:1000 | R |
| 44-488 | Vav3 (Y173) | ThermoFish | 1:1000 | R |
| 2479 | VEGF Receptor 2 (55B11) | CellSig | 1:200 | RmAb |
| 2478 | VEGFR 2 (Y1175) (19A10) | CellSig | 1:50 | RmAb |
| 2471 | VEGFR 2 (Y951) | CellSig | 1:50 | R |
| 2474 | VEGFR 2 (Y996) | CellSig | 1:100 | R |
| 5741 | Vimentin (D21H3) | CellSig | 1:200 | RmAb |
| 2530 | Wnt5a/B (C27E8) | CellSig | 1:100 | RmAb |
| 13008 | YAP (S127) (D9W2I) | CellSig | 1:100 | RmAb |
| 2701 | Zap-70 (Y319)/Syk (Y352) | CellSig | 1:400 | R |
