# Supplementary material for: Acquisition of Resistance to RAS Inhibition Is Associated with the Upregulation of Macropinocytosis through Both PI3K-Dependent and -Independent Signaling
Source: Cancer Res Commun. 2026 Jul 28;6(7):1794–813. doi: 10.1158/2767-9764.CRC-25-0731 (PMC13410306; doi:10.1158/2767-9764.CRC-25-0731)
Supplement: Figure S1 — KRAS suppression results in a transient reduction in macropinocytosis [file crc-25-0731_figure_s1_suppsf1.pdf]

Figure S1

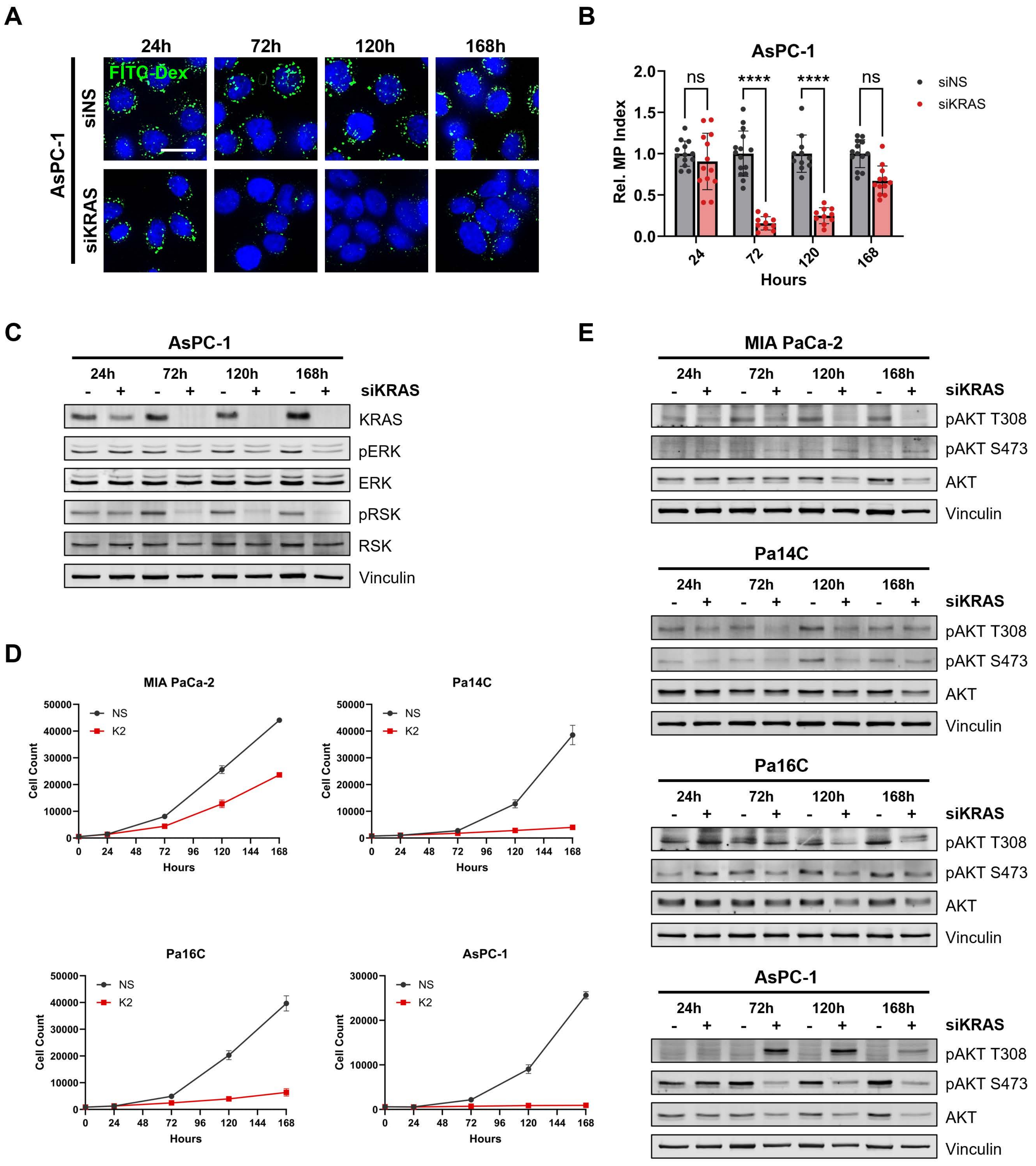

A
24h
72h
120h
168h
FITC-Dex
siNS
siKRAS
AsPC-1
B
AsPC-1
Rel. MP Index
2.0
1.5
1.0
0.5
0.0
ns
****
****
ns
siNS
siKRAS
24
72
120
168
Hours
C
AsPC-1
24h
72h
120h
168h
- + - + - + - +
siKRAS
KRAS
pERK
ERK
pRSK
RSK
Vinculin
D
MIA PaCa-2
Pa14C
Pa16C
AsPC-1
NS
K2
Cell Count
50000
40000
30000
20000
10000
0
0 24 48 72 96 120 144 168
Hours
E
MIA PaCa-2
Pa14C
Pa16C
AsPC-1
24h
72h
120h
168h
- + - + - + - +
siKRAS
pAKT T308
pAKT S473
AKT
Vinculin

Supplementary Figure S1. KRAS suppression results in a transient reduction in macropinocytosis. (A) Representative images of macropinosomes labeled with FITC-dextran (green) and nuclear DAPI stain (blue) in KRAS-mutant AsPC-1 cells transiently transfected with an siRNA oligonucleotide (10 nM) against KRAS (siKRAS) or a non-specific control (siNS) for indicated timepoints. Images are representative of ten fields of view analyzed in each of three independent experiments. Scale bar, 20 μm. **(B)** Quantification of (A), in which the total area of macropinosomes (FITC-dextran immunofluorescence) was calculated and normalized to cell number (Macropinocytic (MP) Index). Relative MP Index is plotted, with each individual data point representing one field containing at least ten analyzed cells. Data are presented as the mean ± SD of one experiment that is representative of three independent experiments. ***$\rho < 0.001$ and ****$\rho < 0.0001$, by the unpaired Student *t*-test, comparing against NS. ns, not significant. **(C)** Immunoblots of AsPC-1 cells treated with siNS and siKRAS as in (A). Vinculin levels were used to monitor equivalent total protein loading. Blots are representative of three independent experiments. **(D)** KRAS-mutant PDAC cells transiently transfected with an siRNA oligonucleotide (10 nM) against KRAS (siKRAS) or a non-specific control (siNS) for indicated timepoints and viability was assessed at indicated timepoints. Each data point represents the mean ± SEM for three biological replicates. **(E)** Immunoblots of indicated KRAS-mutant PDAC cell lines treated with siNS and siKRAS as in (A). Vinculin levels were used to monitor equivalent total protein loading. Blots are representative of three independent experiments.
