# Supplementary material for: Acquisition of Resistance to RAS Inhibition Is Associated with the Upregulation of Macropinocytosis through Both PI3K-Dependent and -Independent Signaling
Source: Cancer Res Commun. 2026 Jul 28;6(7):1794–813. doi: 10.1158/2767-9764.CRC-25-0731 (PMC13410306; doi:10.1158/2767-9764.CRC-25-0731)
Supplement: Figure S2 — Prolonged RAS pathway inhibition in KRAS-mutant PDAC cells results in increased macropinocytosis [file crc-25-0731_figure_s2_suppsf2.pdf]

Figure S2

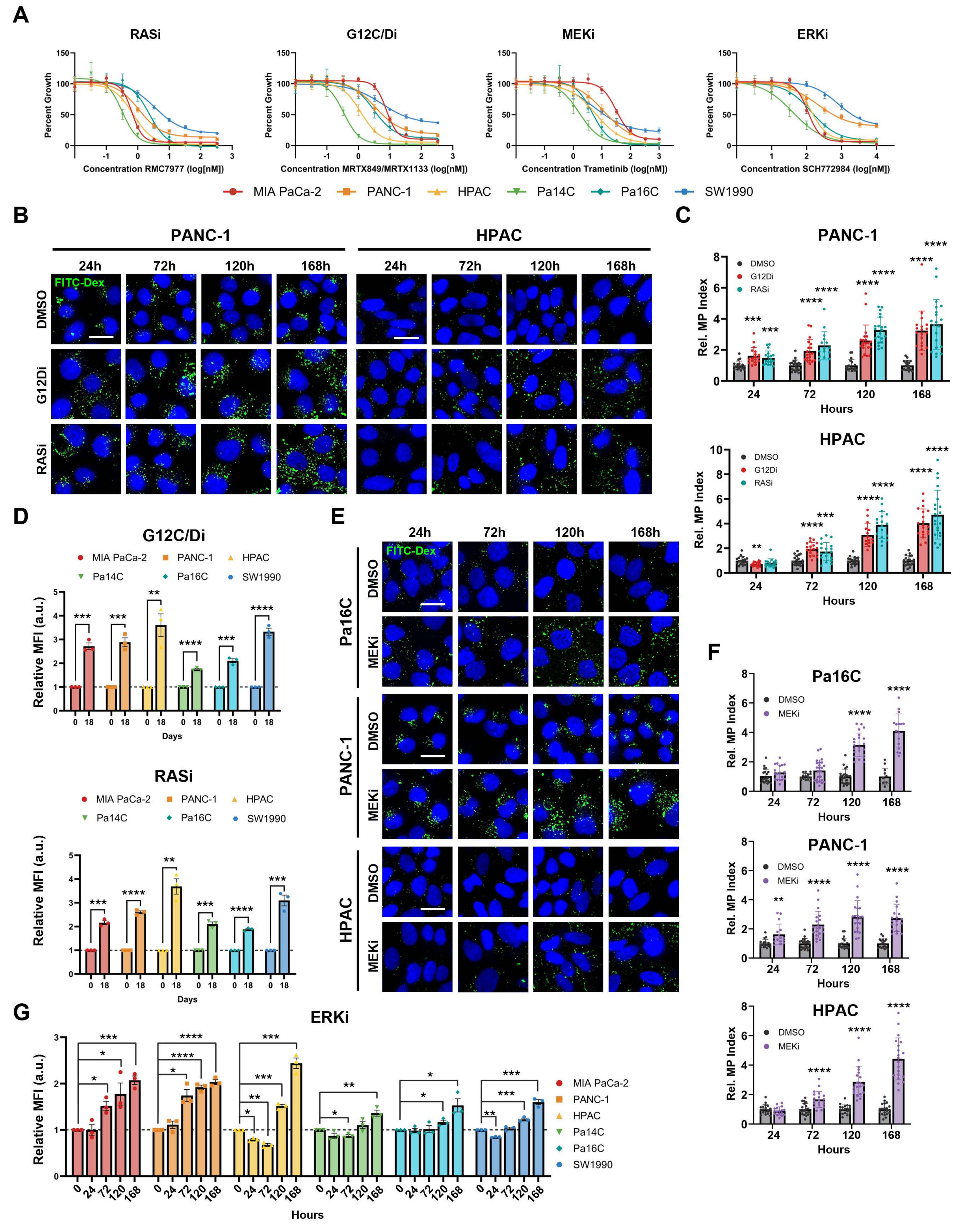

A
RASi
G12C/Di
MEKi
ERKi
Percent Growth
Concentration RMC7977 (log[nM])
Concentration MRTX849/MRTX1133 (log[nM])
Concentration Trametinib (log[nM])
Concentration SCH772984 (log[nM])
MIA PaCa-2
PANC-1
HPAC
Pa14C
Pa16C
SW1990
B
PANC-1
HPAC
24h
72h
120h
168h
FITC-Dex
DMSO
G12Di
RASi
C
PANC-1
HPAC
Rel. MP Index
Hours
D
G12C/Di
RASi
Relative MFI (a.u.)
Days
E
Pa16C
MEKi
F
G
ERKi
Hours

Supplementary Figure S2. Prolonged RAS pathway inhibition in KRAS-mutant PDAC cells results in increased macropinocytosis. (A) Cell viability growth curves of $KRAS^{G12C}$- or $KRAS^{G12D}$-mutant PDAC cell lines treated for five days with RMC-7977 (RASi), MRTX849 (G12Ci), MRTX1133 (G12Di), trametinib (MEKi), or SCH772984 (ERKi). Data are presented as the mean ± SEM of three independent experiments. **(B)** Representative images of macropinosomes labeled with FITC-dextran (green) and nuclear DAPI stain (blue) in indicated KRAS-mutant PDAC cells treated with DMSO or a GI_{60} dose of MRTX1133 (G12Di) or RMC-7977 (RASi). Images are representative of ten fields of view analyzed in each of three independent experiments. Scale bar, 20 μm. **(C)** Quantification of (B), in which Relative (Rel.) MP Index is plotted, with each individual data point representing one field containing at least ten analyzed cells. Data for PANC-1 and HPAC are presented as the mean ± SD of one experiment that is representative of three independent experiments. $^{**}\rho < 0.01$, and $^{***}\rho < 0.001$ and $^{****}\rho < 0.0001$, by the unpaired Student's *t*-test, comparing against DMSO. **(D)** Macropinocytosis was measured via flow cytometry in indicated KRAS-mutant PDAC cell lines that were treated with a GI_{60}-dose of MRTX849 (G12Ci), MRTX1133 (G12Di), or RMC-7977 (RASi) for 18 days. Macropinocytosis was quantified via TMR dextran labeling. Data are presented as the mean ± SEM of three independent experiments. $^{**}\rho < 0.01$, $^{***}\rho < 0.001$, and $^{****}\rho < 0.0001$, by the unpaired Student *t*-test, comparing against DMSO. **(E)** Representative images of macropinosomes labeled with FITC-dextran (green) and nuclear DAPI stain (blue) in indicated KRAS-mutant PDAC cells treated with DMSO or a GI_{60} dose of trametinib (MEKi). Images are representative of ten fields of view analyzed in each of three independent experiments. Scale bar, 20 μm. **(F)** Quantification of (E), in which

Relative MP Index is plotted, with each individual data point representing one field containing at least ten analyzed cells. Data for Pa16C, PANC-1, and HPAC cell lines are presented as the mean +/- SD of one experiment that is representative of three independent experiments. **$\rho < 0.01$, and ****$\rho < 0.0001$, by the unpaired Student's *t*-test, comparing against DMSO. **(G)** Macropinocytosis was measured via flow cytometry in indicated KRAS-mutant PDAC cell lines that were treated with a GI_{60}-dose of SCH778942 (ERKi). Macropinocytosis was quantified via TMR dextran labeling. Data are presented as the mean ± SEM of three independent experiments. *$\rho < 0.05$, **$\rho < 0.01$, ***$\rho < 0.001$, and ****$\rho < 0.0001$, by the unpaired Student's *t*-test, comparing against DMSO.
