# Supplementary material for: Acquisition of Resistance to RAS Inhibition Is Associated with the Upregulation of Macropinocytosis through Both PI3K-Dependent and -Independent Signaling
Source: Cancer Res Commun. 2026 Jul 28;6(7):1794–813. doi: 10.1158/2767-9764.CRC-25-0731 (PMC13410306; doi:10.1158/2767-9764.CRC-25-0731)
Supplement: Figure S3 — Cell lines with acquired resistance to RAS inhibitors exhibit a flattened cellular morphology [file crc-25-0731_figure_s3_suppsf3.pdf]

Figure S3

A

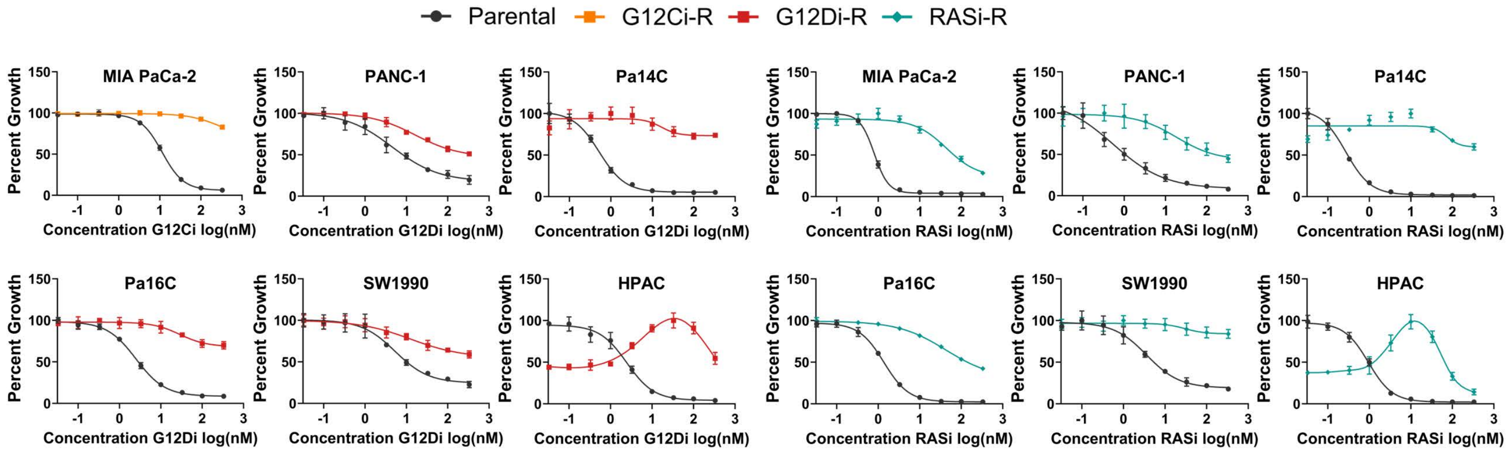

Parental
G12Ci-R
G12Di-R
RASi-R
MIA PaCa-2
PANC-1
Pa14C
Pa16C
SW1990
HPAC
Percent Growth
Concentration G12Ci log(nM)
Concentration G12Di log(nM)
Concentration RASi log(nM)

B

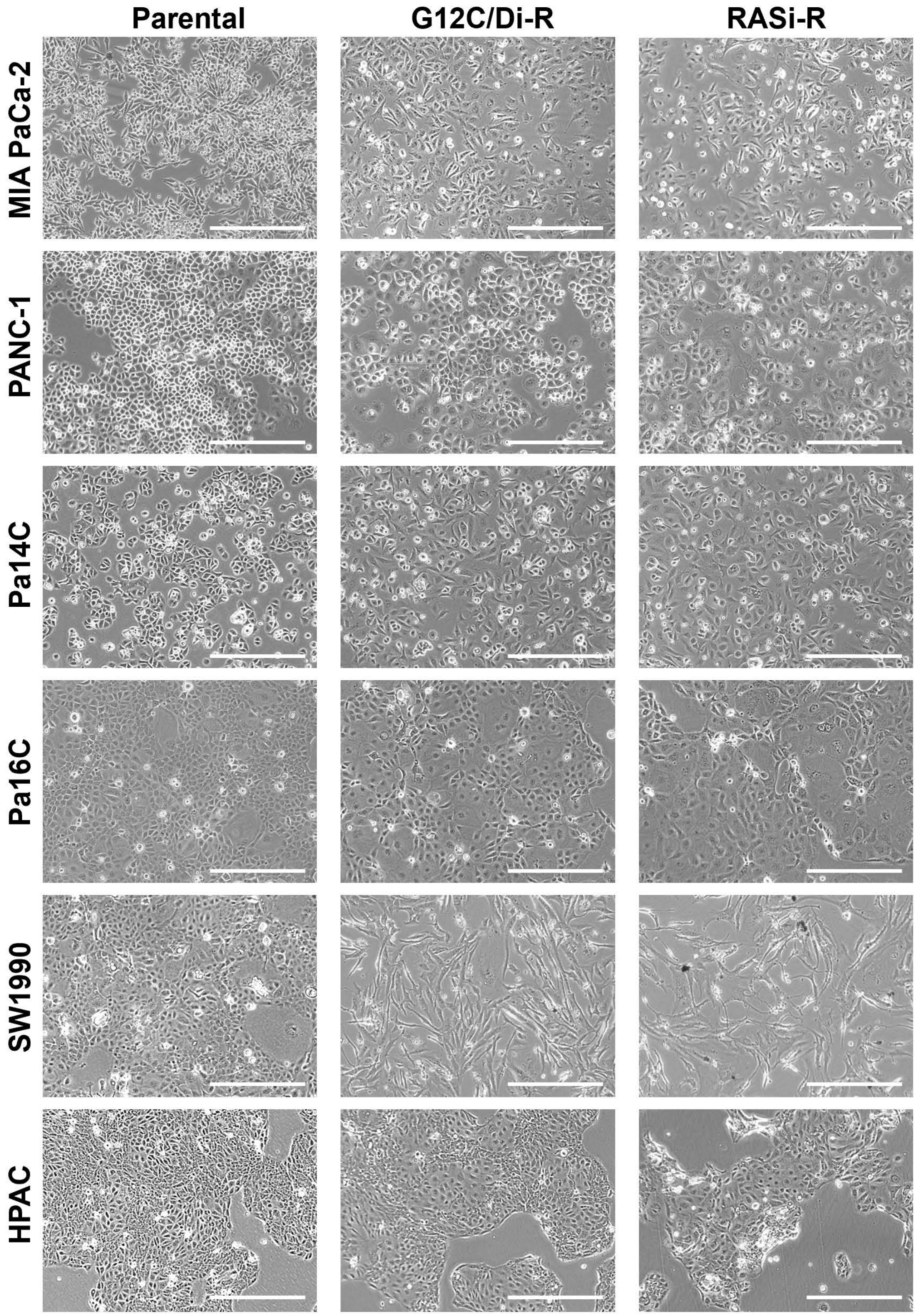

Parental
G12C/Di-R
RASi-R
MIA PaCa-2
PANC-1
Pa14C
Pa16C
SW1990
HPAC

Supplementary Figure S3. Cell lines with acquired resistance to RAS inhibitors exhibit a flattened cellular morphology. (A) Cell viability growth curves of matched parental and either MRTX849- (G12Ci), MRTX1133- (G12Di), or RMC-7977- (RASi) resistant (R) PDAC cell lines treated for 120 hours with an indicated RAS inhibitor (G12Ci, G12Di, or RASi). Data are presented as the mean ± SEM of three independent experiments. **(B)** Representative wide-field images of indicated parental or MRTX849- (G12Ci), MRTX1133-(G12Di), or RMC7797- (RASi) resistant (R) KRAS-mutant PDAC cell lines. Images are representative of ten fields of view. Scale bar, 500 μm.
