# Supplementary material for: Acquisition of Resistance to RAS Inhibition Is Associated with the Upregulation of Macropinocytosis through Both PI3K-Dependent and -Independent Signaling
Source: Cancer Res Commun. 2026 Jul 28;6(7):1794–813. doi: 10.1158/2767-9764.CRC-25-0731 (PMC13410306; doi:10.1158/2767-9764.CRC-25-0731)
Supplement: Figure S4 — Changes to the total proteome following RASi treatment and upon the acquisition of RASi resistance [file crc-25-0731_figure_s4_suppsf4.pdf]

Figure S4

A

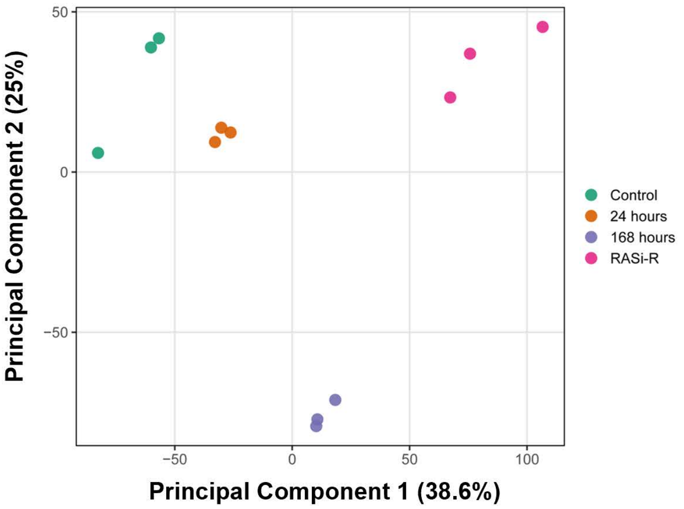
Principal Component 2 (25%)
Principal Component 1 (38.6%)
Control
24 hours
168 hours
RASi-R

B

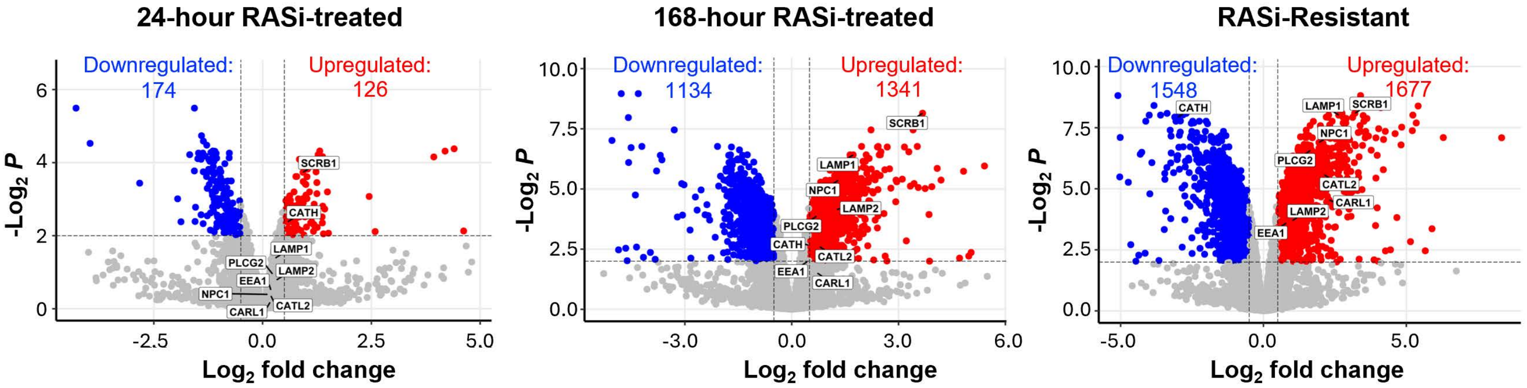
24-hour RASi-treated
Downregulated: 174
Upregulated: 126
168-hour RASi-treated
Downregulated: 1134
Upregulated: 1341
RASi-Resistant
Downregulated: 1548
Upregulated: 1677
-Log2 P
Log2 fold change
SCRB1
CATH
LAMP1
PLCG2
LAMP2
EEA1
NPC1
CATL2
CARL1

Supplementary Figure S4. Changes to the total proteome following RASi treatment and upon the acquisition of RASi resistance. (A) Principal component analysis of $\log_2$ transformed, median-centered protein quantities matrix. **(B)** Differentially expressed proteins following 24 hours RASi (RMC-7977, 3.3 nM), 168 hours RASi (RMC-7977, 3.3 nM), and in a cell line that has acquired RASi-resistance. RAS-regulated proteins ($\rho < 0.05$, 24 hours) are annotated by blue (down-regulated) or red (up-regulated) points.
