# Supplementary material for: Acquisition of Resistance to RAS Inhibition Is Associated with the Upregulation of Macropinocytosis through Both PI3K-Dependent and -Independent Signaling
Source: Cancer Res Commun. 2026 Jul 28;6(7):1794–813. doi: 10.1158/2767-9764.CRC-25-0731 (PMC13410306; doi:10.1158/2767-9764.CRC-25-0731)
Supplement: Figure S5 — RAS inhibitor treatment enhances the sensitivity of PDAC cell lines to albumin-bound nab-paclitaxel but not free paclitaxel [file crc-25-0731_figure_s5_suppsf5.pdf]

Figure S5

A

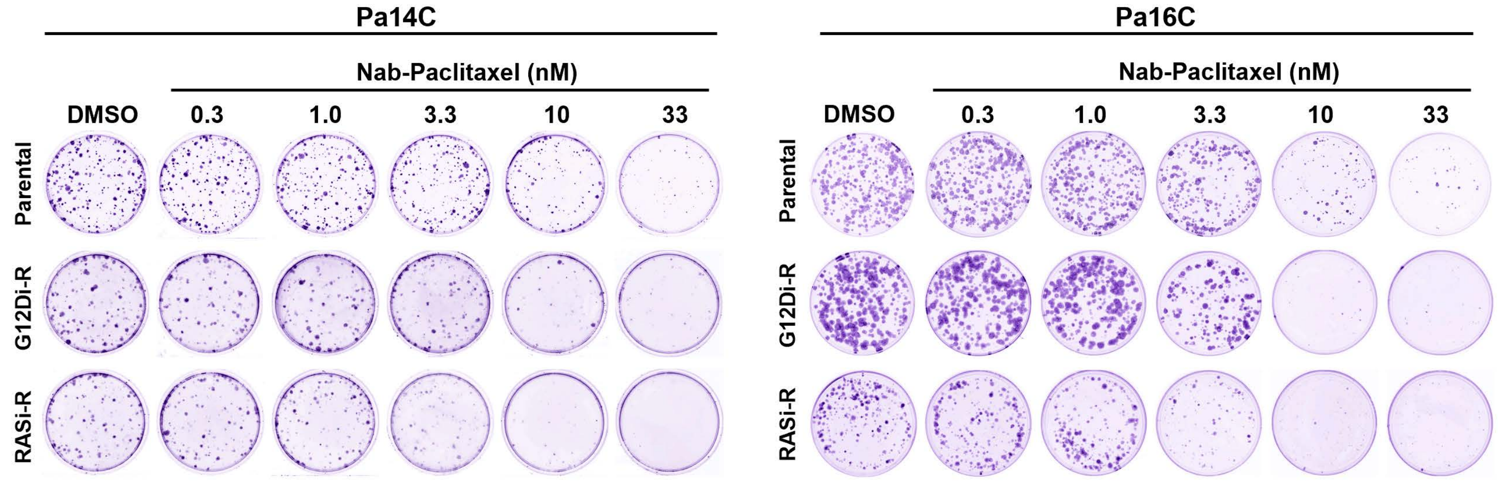
Pa14C
Nab-Paclitaxel (nM)
DMSO
0.3
1.0
3.3
10
33
Parental
G12Di-R
RASi-R
Pa16C
Nab-Paclitaxel (nM)
DMSO
0.3
1.0
3.3
10
33
Parental
G12Di-R
RASi-R

B

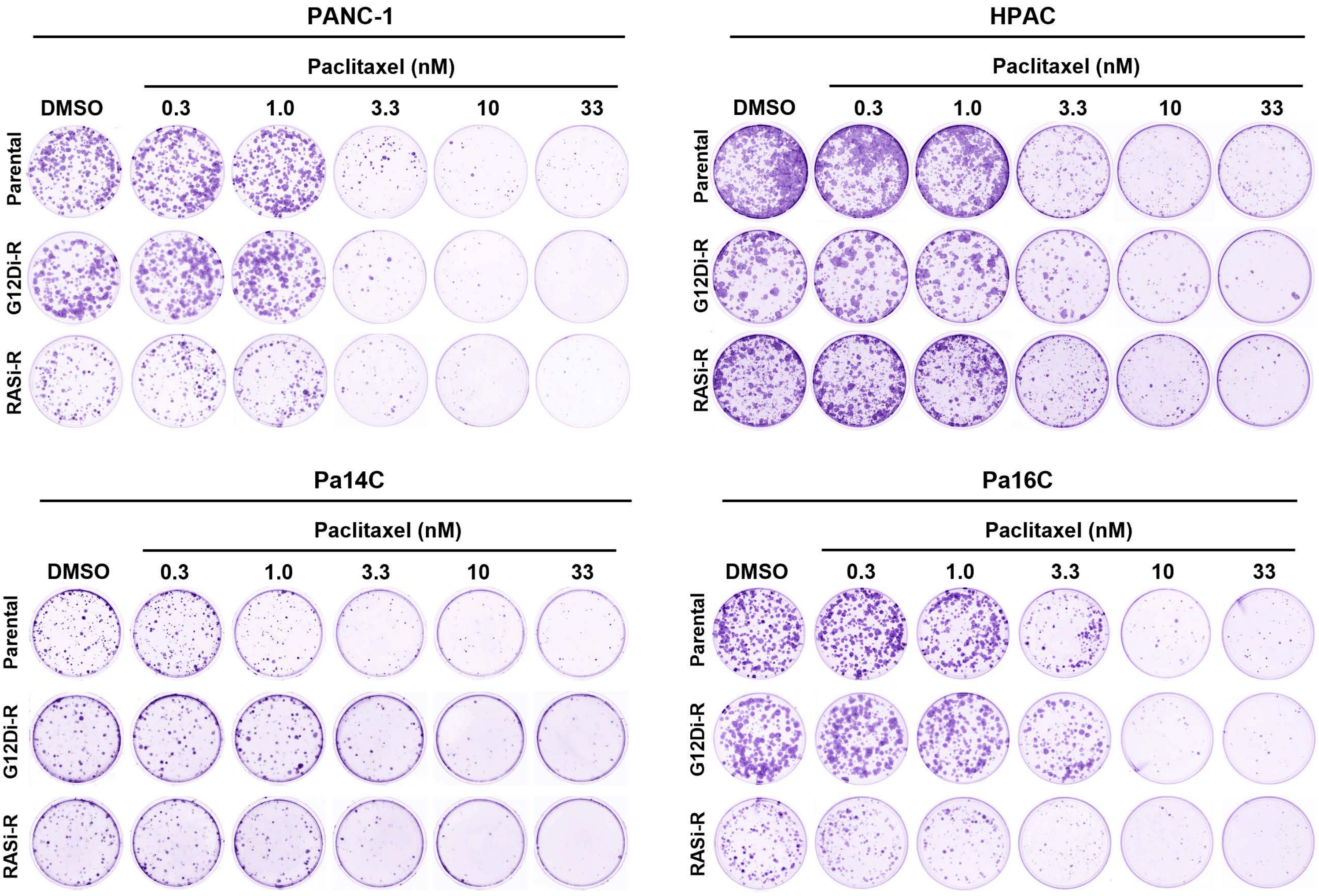
PANC-1
Paclitaxel (nM)
DMSO
0.3
1.0
3.3
10
33
Parental
G12Di-R
RASi-R
HPAC
Paclitaxel (nM)
DMSO
0.3
1.0
3.3
10
33
Parental
G12Di-R
RASi-R
Pa14C
Paclitaxel (nM)
DMSO
0.3
1.0
3.3
10
33
Parental
G12Di-R
RASi-R
Pa16C
Paclitaxel (nM)
DMSO
0.3
1.0
3.3
10
33
Parental
G12Di-R
RASi-R

Supplementary Figure S5. RAS inhibitor treatment enhances the sensitivity of PDAC cell lines to albumin-bound nab-paclitaxel but not free paclitaxel. (A) Representative images of clonogenic growth assays following treatment of matched parental and either MRTX1133- (G12Di) or RMC-7977- (RASi) resistant (R) Pa14C or Pa16C cell lines treated with nab-paclitaxel for 12-16 days. Quantification of these images is represented in Fig. 4D. **(B)** Representative images of clonogenic growth assays following treatment of matched parental and either MRTX1133- (G12Di) or RMC-7977- (RASi) resistant (R) PDAC cell lines treated with paclitaxel for 12-16 days. Quantification of these images is represented in Fig. 4D.
