# Supplementary material for: Acquisition of Resistance to RAS Inhibition Is Associated with the Upregulation of Macropinocytosis through Both PI3K-Dependent and -Independent Signaling
Source: Cancer Res Commun. 2026 Jul 28;6(7):1794–813. doi: 10.1158/2767-9764.CRC-25-0731 (PMC13410306; doi:10.1158/2767-9764.CRC-25-0731)
Supplement: Figure S6 — MEK inhibitor treatment enhances the sensitivity of PDAC cell lines to albumin-bound nab-paclitaxel but not free paclitaxel [file crc-25-0731_figure_s6_suppsf6.pdf]

# Figure S6

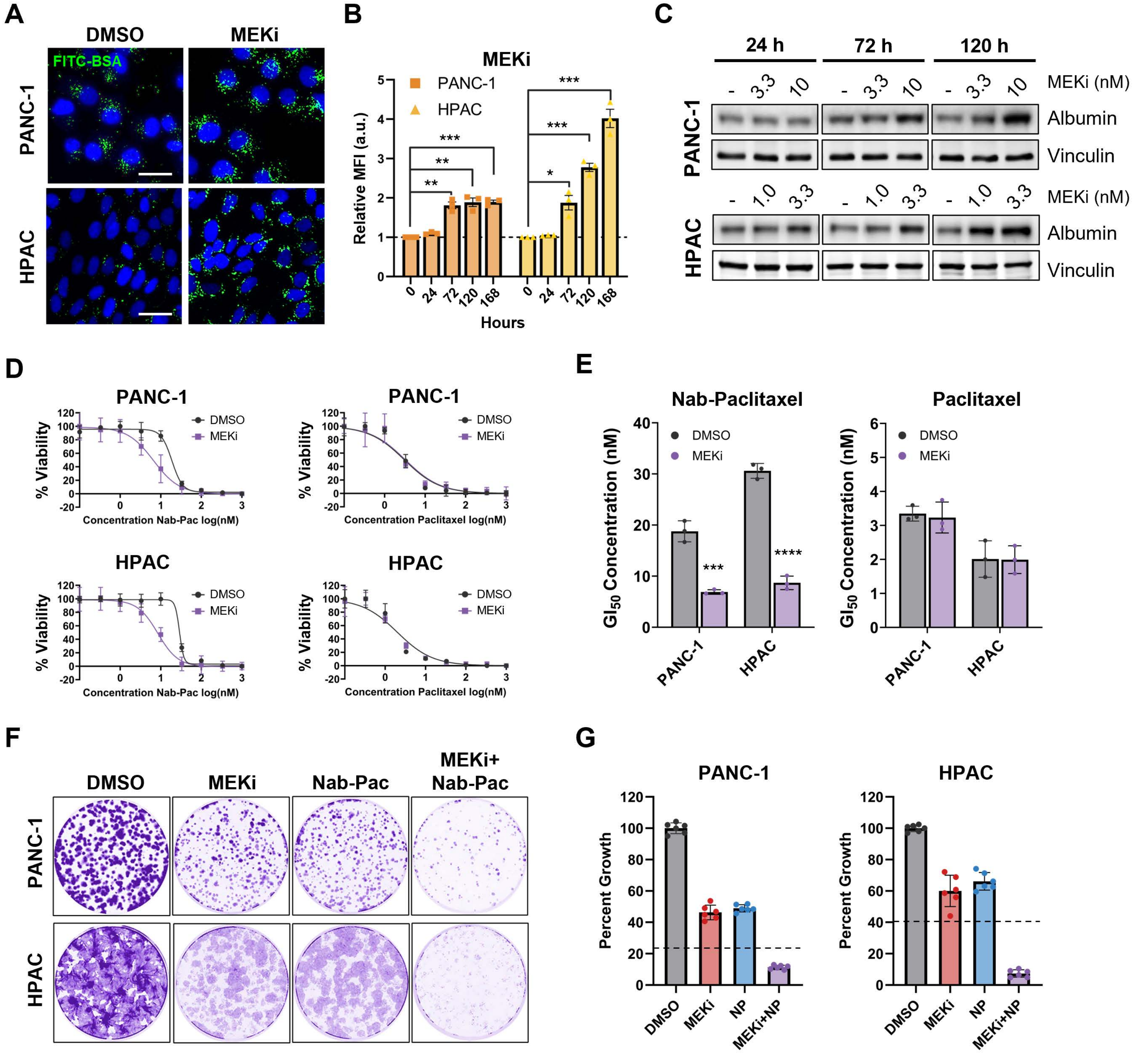

A
DMSO
MEKi
FITC-BSA
PANC-1
HPAC
B
MEKi
PANC-1
HPAC
Relative MFI (a.u.)
Hours
0
24
72
120
168
C
24 h
72 h
120 h
MEKi (nM)
Albumin
Vinculin
D
PANC-1
HPAC
% Viability
DMSO
MEKi
Concentration Nab-Pac log(nM)
Concentration Paclitaxel log(nM)
E
Nab-Paclitaxel
Paclitaxel
GI50 Concentration (nM)
F
DMSO
MEKi
Nab-Pac
MEKi+
Nab-Pac
G
PANC-1
HPAC
Percent Growth
DMSO
MEKi
NP
MEKi+NP

**Supplementary Figure S6. MEK inhibitor treatment enhances the sensitivity of PDAC cell lines to albumin-bound nab-paclitaxel but not free paclitaxel. (A)** Representative images of macropinosomes labeled with FITC-BSA (green) and nuclear DAPI stain (blue) in indicated PDAC cells treated with DMSO or MEKi (trametinib: 10 nM in PANC-1, 3.3 nM in HPAC). Images are representative of ten fields of view analyzed in each of two independent experiments. Scale bar, 20 μm. **(B)** Macropinocytosis was measured via flow cytometry in indicated KRAS-mutant PDAC cell lines that were treated with MEKi (trametinib: 10 nM in PANC-1, 3.3 nM in HPAC) for 24, 72, 120, or 168 hours. Macropinocytosis was quantified via FITC-BSA labeling. Data are presented as the mean ± SEM of three independent experiments. *$\rho < 0.05$, **$\rho < 0.01$, ***$\rho < 0.001$, and ****$\rho < 0.0001$, by the unpaired Student's *t*-test, comparing against DMSO. **(C)** Immunoblot for human serum albumin (0.5% w/v for 1 hour before lysis) following treatment of indicated PDAC cell lines with trametinib (MEKi) at indicated concentrations and durations. **(D)** Relative viability following a five-day growth assay of indicated PDAC cell lines treated with a dose response of nab-paclitaxel (Nab-Pac) in the presence of absence of MEKi (trametinib, 10 nM in PANC-1, 3.3 nM in HPAC). Data are presented as the mean ± SEM of three independent experiments. **(E)** $GI_{50}$ values from data plotted in (D) for nab-paclitaxel treated lines (left) and paclitaxel treated lines (right). ***$\rho < 0.001$, and ****$\rho < 0.0001$ by the unpaired Student's *t*-test, comparing against the parental cell line. **(F)** Representative images of clonogenic growth assays following treatment of indicated PDAC cell lines with trametinib (MEKi), nab-paclitaxel (Nab-Pac), or the combination for 12-16 days. **(G)** Relative colony number from experiment described in (F). Data are presented as the mean ± SEM of six independent experiments and normalized to DMSO-

treated condition which is set to 100% growth. Dashed line represents the expected effect of additive interaction between MEKi and Nab-Pac, based on Bliss Independence.
