# Supplementary material for: Acquisition of Resistance to RAS Inhibition Is Associated with the Upregulation of Macropinocytosis through Both PI3K-Dependent and -Independent Signaling
Source: Cancer Res Commun. 2026 Jul 28;6(7):1794–813. doi: 10.1158/2767-9764.CRC-25-0731 (PMC13410306; doi:10.1158/2767-9764.CRC-25-0731)
Supplement: Figure S7 — RPPA analysis of PDAC cell line panel treated with RAS ERK MAPK inhibitors for 24 to 168 hours [file crc-25-0731_figure_s7_suppsf7.pdf]

Figure S7

A

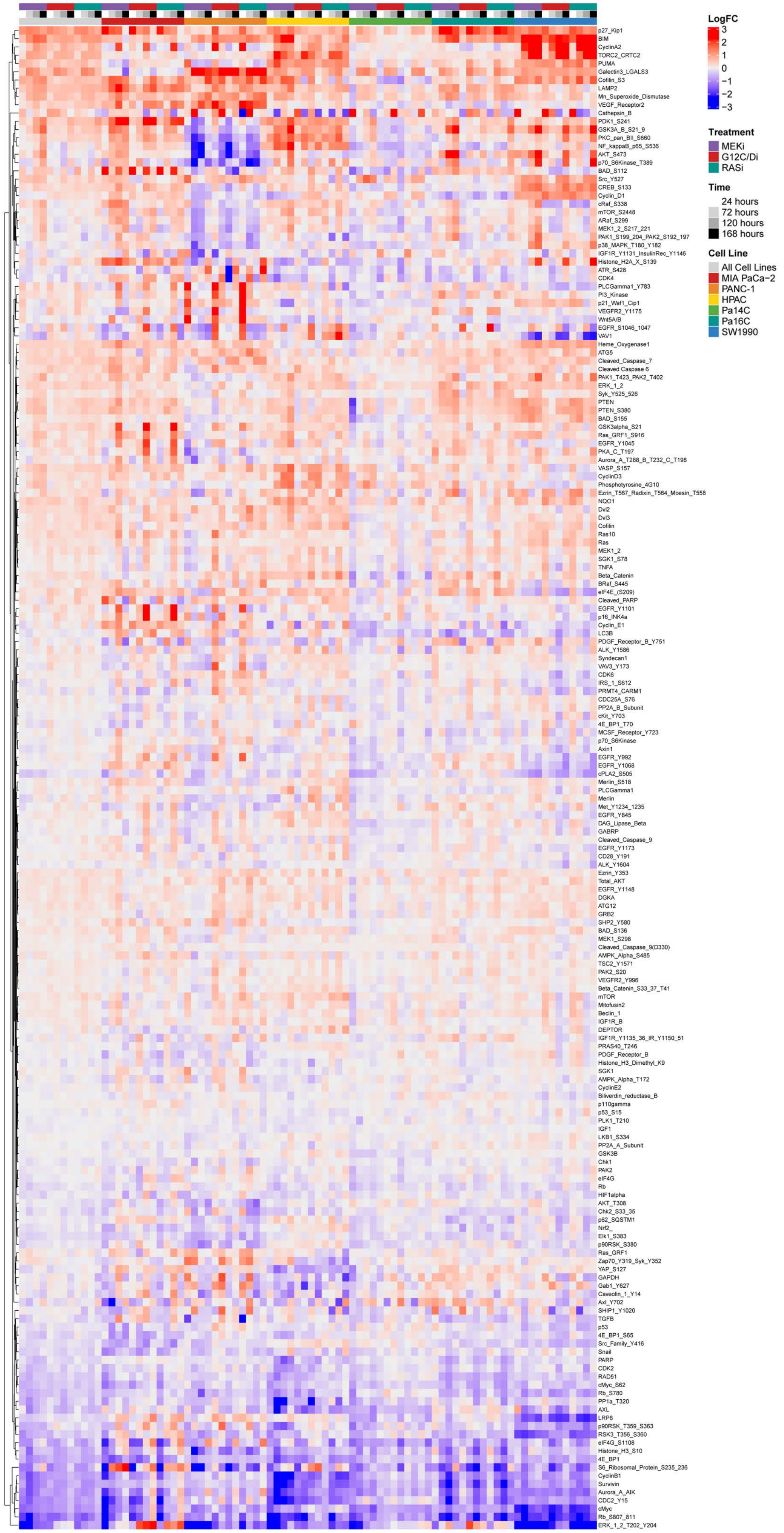

LogFC
3
2
1
0
−1
−2
−3
Treatment
MEKi
G12C/Di
RASi
Time
24 hours
72 hours
120 hours
168 hours
Cell Line
All Cell Lines
MIA PaCa−2
PANC-1
HPAC
Pa14C
Pa16C
SW1990
p27_Kip1
BIM
CyclinA2
TORC2_CRTC2
PUMA
Galectin3_LGALS3
Cofilin_S3
LAMP2
Mn_Superoxide_Dismutase
VEGF_Receptor2
Cathepsin_B
PDK1_S241
GSK3A_B_S21_9
PKC_pan_BII_S660
NF_kappaB_p65_S536
AKT_S473
p70_S6Kinase_T389
BAD_S112
Src_Y527
CREB_S133
Cyclin_D1
cRaf_S338
mTOR_S2448
ARaf_S299
MEK1_2_S217_221
PAK1_S199_204_PAK2_S192_197
p38_MAPK_T180_Y182
IGF1R_Y1131_InsulinRec_Y1146
Histone_H2A_X_S139
ATR_S428
CDK4
PLCGamma1_Y783
PI3_Kinase
p21_Waf1_Cip1
VEGFR2_Y1175
Wnt5A/B
EGFR_S1046_1047
VAV1
Heme_Oxygenase1
ATG5
Cleaved_Caspase_7
Cleaved Caspase 6
PAK1_T423_PAK2_T402
ERK_1_2
Syk_Y525_526
PTEN
PTEN_S380
BAD_S155
GSK3alpha_S21
Ras_GRF1_S916
EGFR_Y1045
PKA_C_T197
Aurora_A_T288_B_T232_C_T198
VASP_S157
CyclinD3
Phosphotyrosine_4G10
Ezrin_T567_Radixin_T564_Moesin_T558
NQO1
Dvl2
Dvl3
Cofilin
Ras10
Ras
MEK1_2
SGK1_S78
TNFA
Beta_Catenin
BRaf_S445
eIF4E_(S209)
Cleaved_PARP
EGFR_Y1101
p16_INK4a
Cyclin_E1
LC3B
PDGF_Receptor_B_Y751
ALK_Y1586
Syndecan1
VAV3_Y173
CDK6
IRS_1_S612
PRMT4_CARM1
CDC25A_S76
PP2A_B_Subunit
cKit_Y703
4E_BP1_T70
MCSF_Receptor_Y723
p70_S6Kinase
Axin1
EGFR_Y992
EGFR_Y1068
cPLA2_S505
Merlin_S518
PLCGamma1
Merlin
Met_Y1234_1235
EGFR_Y845
DAG_Lipase_Beta
GABRP
Cleaved_Caspase_9
EGFR_Y1173
CD28_Y191
ALK_Y1604
Ezrin_Y353
Total_AKT
EGFR_Y1148
DGKA
ATG12
GRB2
SHP2_Y580
BAD_S136
MEK1_S298
Cleaved_Caspase_9(D330)
AMPK_Alpha_S485
TSC2_Y1571
PAK2_S20
VEGFR2_Y996
Beta_Catenin_S33_37_T41
mTOR
Mitofusin2
Beclin_1
IGF1R_B
DEPTOR
IGF1R_Y1135_36_IR_Y1150_51
PRAS40_T246
PDGF_Receptor_B
Histone_H3_Dimethyl_K9
SGK1
AMPK_Alpha_T172
CyclinE2
Biliverdin_reductase_B
p110gamma
p53_S15
PLK1_T210
IGF1
LKB1_S334
PP2A_A_Subunit
GSK3B
Chk1
PAK2
eIF4G
Rb
HIF1alpha
AKT_T308
Chk2_S33_35
p62_SQSTM1
Nrf2
Elk1_S383
p90RSK_S380
Ras_GRF1
Zap70_Y319_Syk_Y352
YAP_S127
GAPDH
Gab1_Y627
Caveolin_1_Y14
Axl_Y702
SHIP1_Y1020
TGFB
p53
4E_BP1_S65
Src_Family_Y416
Snail
PARP
CDK2
RAD51
cMyc_S62
Rb_S780
PP1a_T320
AXL
LRP6
p90RSK_T359_S363
RSK3_T356_S360
eIF4G_S1108
Histone_H3_S10
4E_BP1
S6_Ribosomal_Protein_S235_236
CyclinB1
Survivin
Aurora_A_AIK
CDC2_Y15
cMyc
Rb_S807_811
ERK_1_2_T202_Y204

Supplementary Figure S7. RPPA analysis of PDAC cell line panel treated with RAS ERK MAPK inhibitors for 24 to 168 hours. Heat map showing median log_2(FC) of the 181 proteins/phosphoproteins evaluated by RPPA in six individual cell lines treated for 24, 72, 120, and 168 hours with DMSO, trametinib (MEKi), MRTX849/MRTX1133 (G12C/Di), or RMC-7977 (RASi). The median of four biological replicates for each drug treatment condition is represented, and log_2(FC) was calculated compared to DMSO control for each time point.
