# Supplementary material for: Acquisition of Resistance to RAS Inhibition Is Associated with the Upregulation of Macropinocytosis through Both PI3K-Dependent and -Independent Signaling
Source: Cancer Res Commun. 2026 Jul 28;6(7):1794–813. doi: 10.1158/2767-9764.CRC-25-0731 (PMC13410306; doi:10.1158/2767-9764.CRC-25-0731)
Supplement: Figure S8 — PDAC cell lines display both PI3K-dependent and -independent signaling supporting macropinocytosis [file crc-25-0731_figure_s8_suppsf8.pdf]

Figure S8

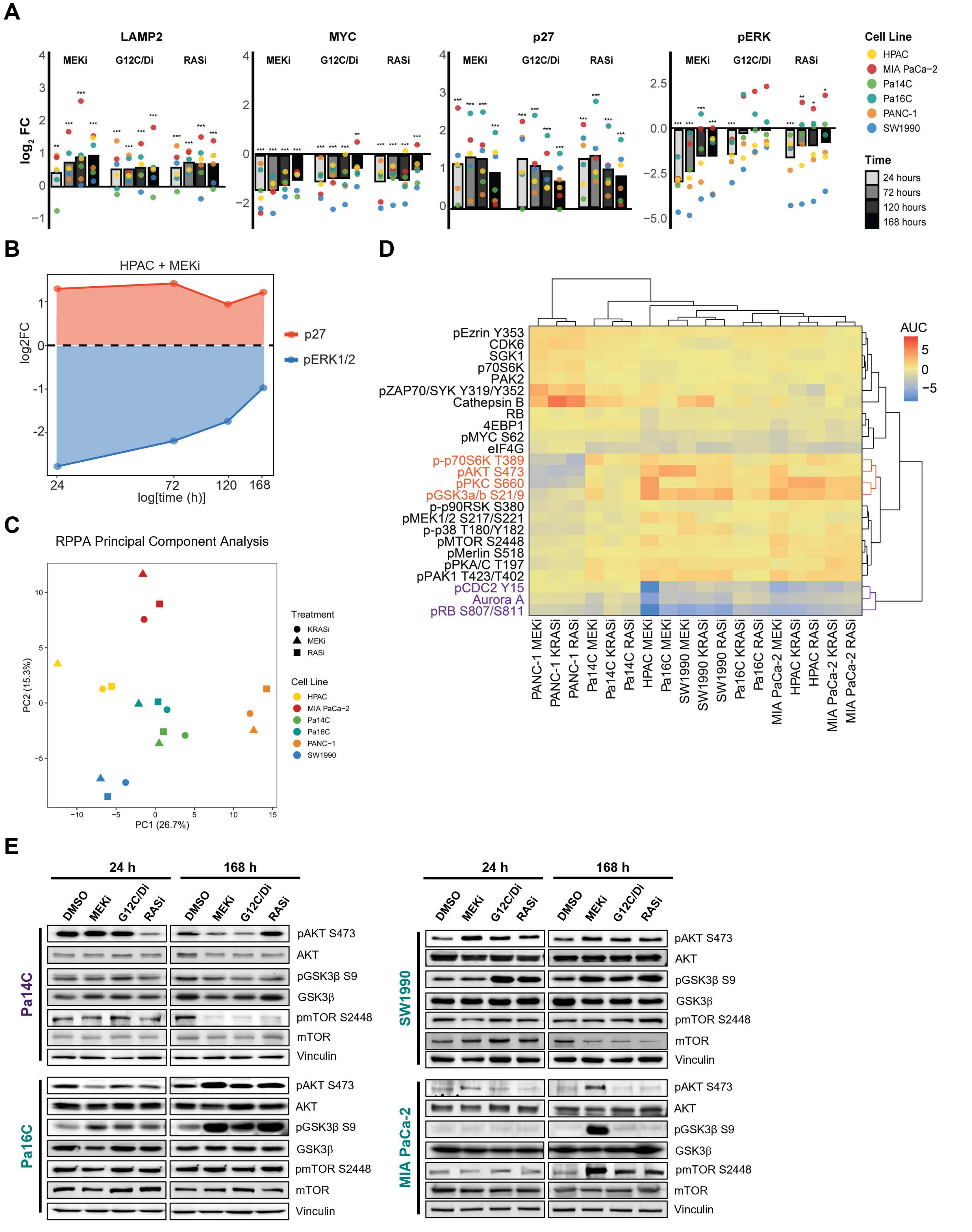
A
LAMP2
MYC
p27
pERK
MEKi
G12C/Di
RASi
log2 FC
Cell Line
HPAC
MIA PaCa-2
Pa14C
Pa16C
PANC-1
SW1990
Time
24 hours
72 hours
120 hours
168 hours
B
HPAC + MEKi
p27
pERK1/2
log2FC
log[time (h)]
C
RPPA Principal Component Analysis
PC1 (26.7%)
PC2 (15.3%)
Treatment
KRASi
MEKi
RASi
D
AUC
pEzrin Y353
CDK6
SGK1
p70S6K
PAK2
pZAP70/SYK Y319/Y352
Cathepsin B
RB
4EBP1
pMYC S62
eIF4G
p-p70S6K T389
pAKT S473
pPKC S660
pGSK3a/b S21/9
p-p90RSK S380
pMEK1/2 S217/S221
p-p38 T180/Y182
pMTOR S2448
pMerlin S518
pPKA/C T197
pPAK1 T423/T402
pCDC2 Y15
Aurora A
pRB S807/S811
PANC-1 MEKi
PANC-1 KRASi
PANC-1 RASi
Pa14C MEKi
Pa14C KRASi
Pa14C RASi
HPAC MEKi
Pa16C MEKi
SW1990 MEKi
SW1990 KRASi
SW1990 RASi
Pa16C KRASi
Pa16C RASi
MIA PaCa-2 MEKi
HPAC KRASi
HPAC RASi
MIA PaCa-2 KRASi
MIA PaCa-2 RASi
E
24 h
168 h
DMSO
MEKi
G12C/Di
RASi
Pa14C
Pa16C
SW1990
MIA PaCa-2
pAKT S473
AKT
pGSK3β S9
GSK3β
pmTOR S2448
mTOR
Vinculin

Supplementary Figure S8. PDAC cell lines display both PI3K-dependent and -independent signaling supporting macropinocytosis. (A) Bar graphs of RPPA log_2(FC) values for selected antibodies comparing all cell lines, treatment conditions, and time points as described in Supplementary Fig. 7. **(B)** Example showing AUC calculation. Using median RPPA log_2(FC) expression data over all time points data was collapsed to a single area under the curve (AUC) value. **(C)** Principal component analysis of log_2 transformed, median-centered (phospho)protein quantities matrix. **(D)** Heat map showing top 25 differential antibodies between Group 1 and Group 2 summarized by AUC. Grouping of cell cycle regulating proteins (pRB, Aurora A, CDC2), where PANC-1 and Pa14C show near 0 AUC and other cell lines depressed AUC. Similarly, p-p70S6K, pAKT, pPKC, and pGSK3a/b are near 0 for PANC-1 and Pa14C but above 0 for other cell lines. **(E)** Immunoblotting of indicated proteins in Pa14C, Pa16C, SW1990 and MIA PaCa-2cells treated identically to RPPA samples with DMSO, MEKi, G12Di, or RASi for 24 or 168 hours.
