# Supplementary material for: Acquisition of Resistance to RAS Inhibition Is Associated with the Upregulation of Macropinocytosis through Both PI3K-Dependent and -Independent Signaling
Source: Cancer Res Commun. 2026 Jul 28;6(7):1794–813. doi: 10.1158/2767-9764.CRC-25-0731 (PMC13410306; doi:10.1158/2767-9764.CRC-25-0731)
Supplement: Figure S9 — Pictilisib treatment impairs growth and PI3K signaling in PDAC cell lines [file crc-25-0731_figure_s9_suppsf9.pdf]

# Figure S9

A

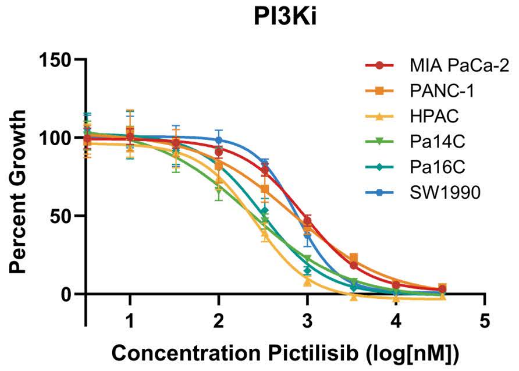
PI3Ki
MIA PaCa-2
PANC-1
HPAC
Pa14C
Pa16C
SW1990
Percent Growth
150
100
50
0
1
2
3
4
5
Concentration Pictilisib (log[nM])

B

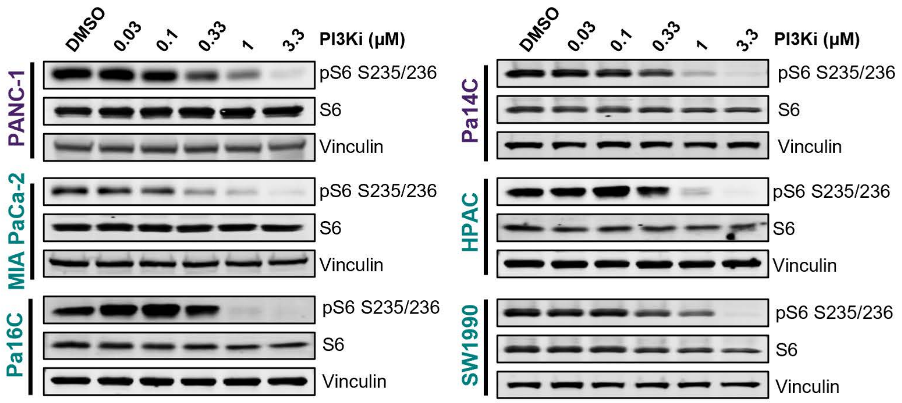
DMSO
0.03
0.1
0.33
1
3.3
PI3Ki (μM)
PANC-1
MIA PaCa-2
Pa16C
Pa14C
HPAC
SW1990
pS6 S235/236
S6
Vinculin

**Supplementary Figure S9. Pictilisib treatment impairs growth and PI3K signaling in PDAC cell lines. (A)** Cell viability growth curves of PDAC cell line panel treated for five days with pictilisib (PI3Ki). Data are presented as the mean ± SEM of three independent experiments. **(B)** Immunoblotting for phospho-S6, total S6 to assess on target activity and vinculin loading control in PDAC cell line panel treated for 24 hours with DMSO or pictilisib (PI3Ki) at increasing doses.
