# Supplementary material for: Acquisition of Resistance to RAS Inhibition Is Associated with the Upregulation of Macropinocytosis through Both PI3K-Dependent and -Independent Signaling
Source: Cancer Res Commun. 2026 Jul 28;6(7):1794–813. doi: 10.1158/2767-9764.CRC-25-0731 (PMC13410306; doi:10.1158/2767-9764.CRC-25-0731)
Supplement: Figure S10 — Activation of the PI3K and FAK pathways results in increased RAC activity in RASi-resistant cell lines [file crc-25-0731_figure_s10_suppsf10.pdf]

Figure S10

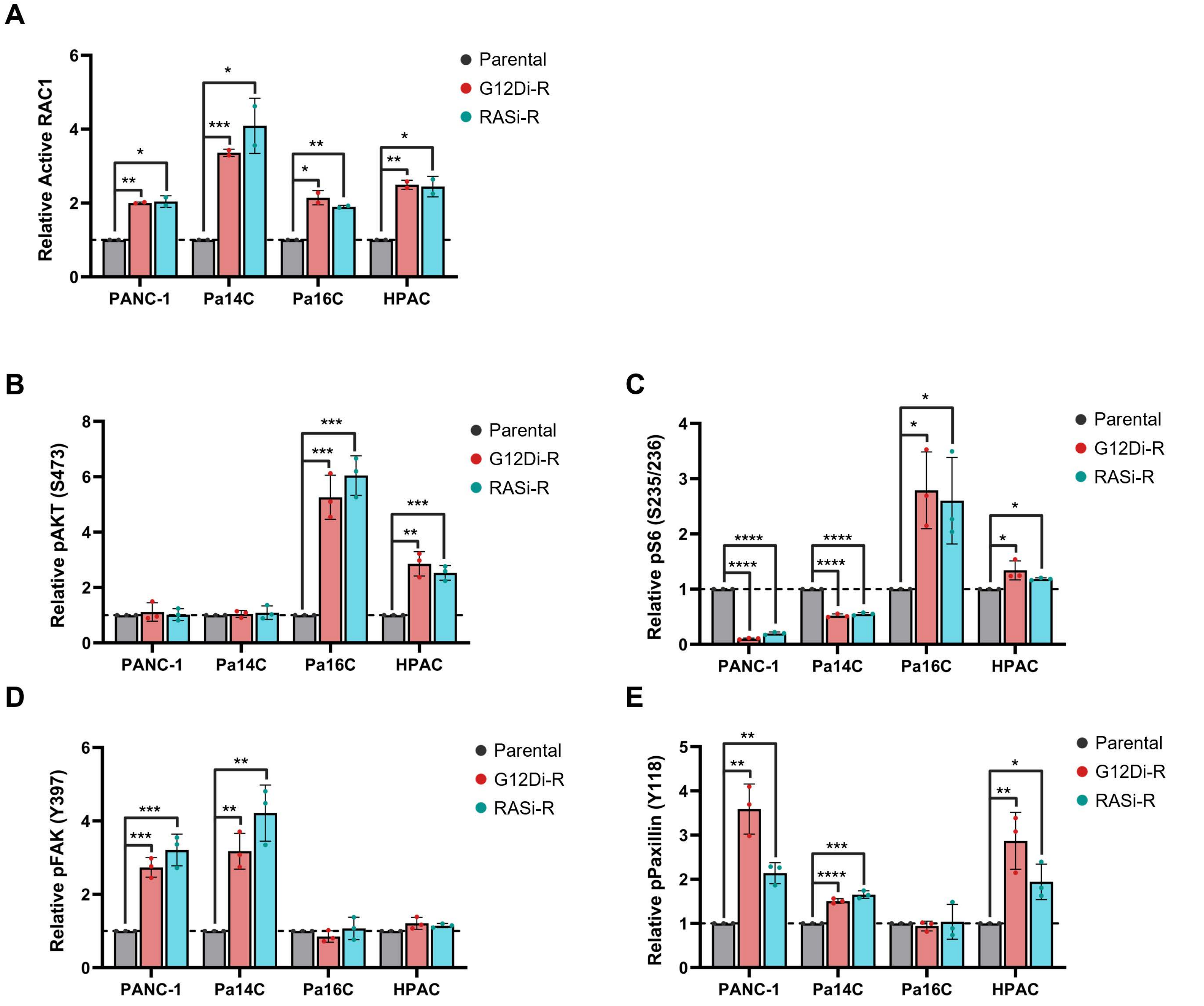
A
Relative Active RAC1
PANC-1
Pa14C
Pa16C
HPAC
Parental
G12Di-R
RASi-R
B
Relative pAKT (S473)
C
Relative pS6 (S235/236)
D
Relative pFAK (Y397)
E
Relative pPaxillin (Y118)

Supplementary Figure S10. Activation of the PI3K and FAK pathways results in increased RAC activity in RASi-resistant cell lines. (A) Densitometry of active RAC1 in KRAS G12D and RAS inhibitor resistant cell lines normalized to their parental counterparts from (immunoblot shown in Fig. 6B). **(B-E)** Densitometry of downstream effectors in PI3K and FAK signaling pathways and analysis of cellular signaling alterations following treatment with pictilisib (PI3Ki) and defactinib (FAKi). Densitometry quantitation of pAKT S473 (B), pS6 S235/236 (C), pFAK Y397 (D), and pPaxillin Y118 (E) normalized to respective total proteins (immunoblots shown in Fig. 6C). Data are presented as the mean ± SEM of three independent experiments. Data are presented as the mean ± SEM of three independent experiments. *$\rho < 0.05$, **$\rho < 0.01$, ***$\rho < 0.001$, and ****$\rho < 0.0001$ by the unpaired Student's *t*-test, comparing against each respective parental line.
