# Supplementary material for: Acquisition of Resistance to RAS Inhibition Is Associated with the Upregulation of Macropinocytosis through Both PI3K-Dependent and -Independent Signaling
Source: Cancer Res Commun. 2026 Jul 28;6(7):1794–813. doi: 10.1158/2767-9764.CRC-25-0731 (PMC13410306; doi:10.1158/2767-9764.CRC-25-0731)
Supplement: Figure S11 — Pictilisib (PI3Ki) treatment decreases PI3K-mediated signaling in PDAC cell lines [file crc-25-0731_figure_s11_suppsf11.pdf]

Figure S11

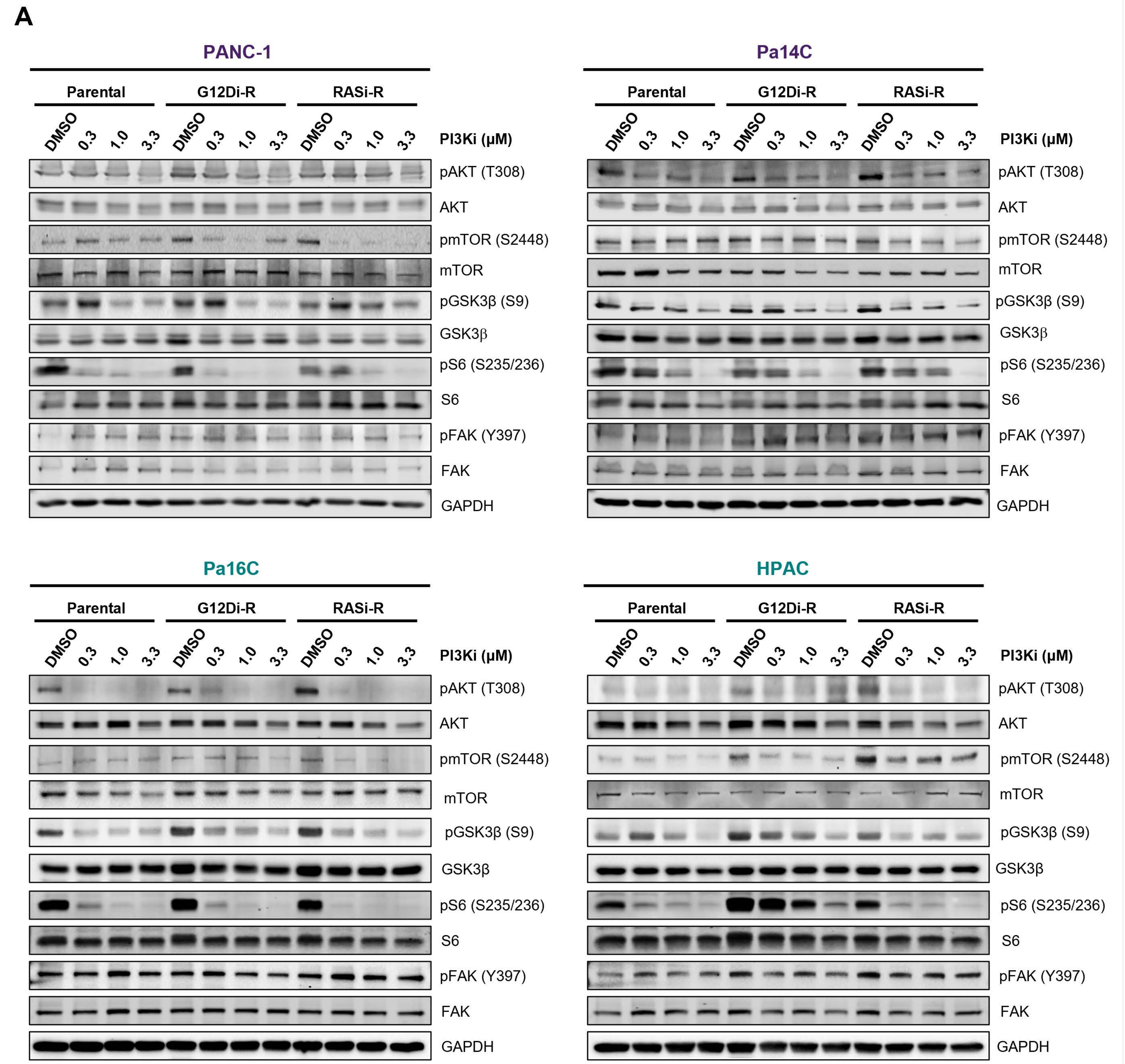
A
PANC-1
Pa14C
Pa16C
HPAC
Parental
G12Di-R
RASi-R
DMSO
0.3
1.0
3.3
PI3Ki (µM)
pAKT (T308)
AKT
pmTOR (S2448)
mTOR
pGSK3β (S9)
GSK3β
pS6 (S235/236)
S6
pFAK (Y397)
FAK
GAPDH

Supplementary Figure S11. Pictilisib (PI3Ki) treatment decreases PI3K-mediated signaling in PDAC cell lines. Immunoblotting for indicated proteins, in parental, G12Di-R, and RASi-R cell line panel treated for 24 hours with DMSO or pictilisib (PI3Ki) at increasing doses.
