# Supplementary material for: Acquisition of Resistance to RAS Inhibition Is Associated with the Upregulation of Macropinocytosis through Both PI3K-Dependent and -Independent Signaling
Source: Cancer Res Commun. 2026 Jul 28;6(7):1794–813. doi: 10.1158/2767-9764.CRC-25-0731 (PMC13410306; doi:10.1158/2767-9764.CRC-25-0731)
Supplement: Figure S12 — Defactinib (FAKi) treatment decreases FAK-mediated signaling and macropinocytosis in PDAC cell lines [file crc-25-0731_figure_s12_suppsf12.pdf]

Figure S12

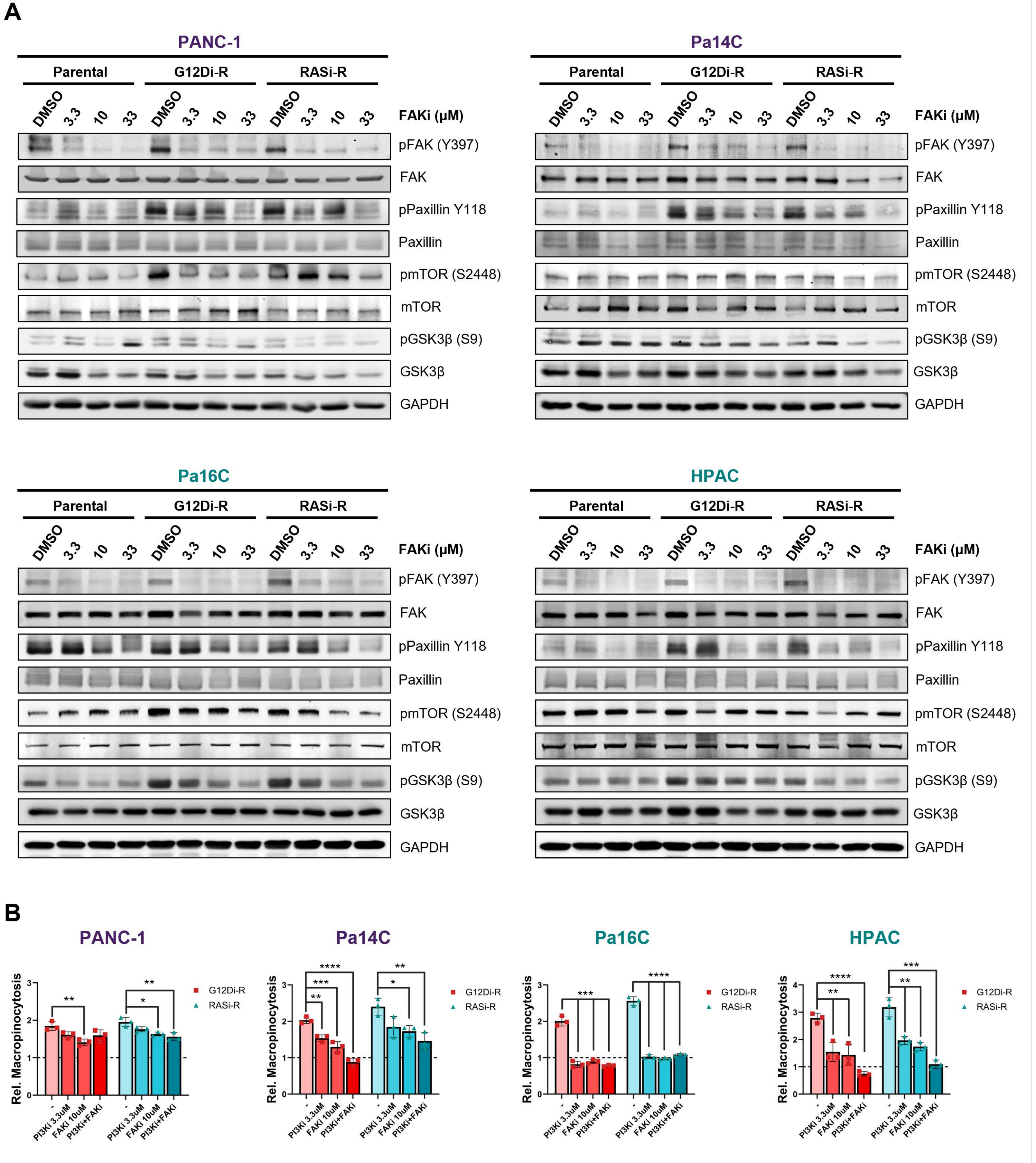

A
PANC-1
Pa14C
Pa16C
HPAC
Parental
G12Di-R
RASi-R
DMSO
3.3
10
33
FAKi (μM)
pFAK (Y397)
FAK
pPaxillin Y118
Paxillin
pmTOR (S2448)
mTOR
pGSK3β (S9)
GSK3β
GAPDH
B
Rel. Macropinocytosis
G12Di-R
RASi-R
-
PI3Ki 3.3uM
FAKi 10uM
PI3Ki+FAKi

Supplementary Figure S12. Defactinib (FAKi) treatment decreases FAK-mediated signaling and macropinocytosis in PDAC cell lines. (A) Immunoblotting for indicated proteins, in parental, G12Di-R, and RASi-R cell line panel treated for 24 hours with DMSO or defactinib (FAKi) at increasing doses. **(B)** Macropinocytosis was measured via flow cytometry in indicated parental, MRTX1133- (G12Di) resistant (R), or RMC-7977- (RASi) resistant (R) KRAS-mutant PDAC cell lines with or without indicated doses of defactinib (FAKi), pictilisib (PI3Ki) or a combination of FAKi and PI3Ki. Macropinocytosis was quantified via TMR dextran labeling. Data are presented as the mean ± SEM of three independent experiments. *$\rho < 0.05$, **$\rho < 0.01$, ***$\rho < 0.001$, and ****$\rho < 0.0001$, by the unpaired Student's *t*-test, comparing each inhibitor treatment against DMSO for each line.
