# Supplementary material for: Acquisition of Resistance to RAS Inhibition Is Associated with the Upregulation of Macropinocytosis through Both PI3K-Dependent and -Independent Signaling
Source: Cancer Res Commun. 2026 Jul 28;6(7):1794–813. doi: 10.1158/2767-9764.CRC-25-0731 (PMC13410306; doi:10.1158/2767-9764.CRC-25-0731)
Supplement: Figure S13 — A subset of RASi-resistant PDAC cell lines activates YAP and/or TAZ to upregulate macropinocytosis [file crc-25-0731_figure_s13_suppsf13.pdf]

Figure S13

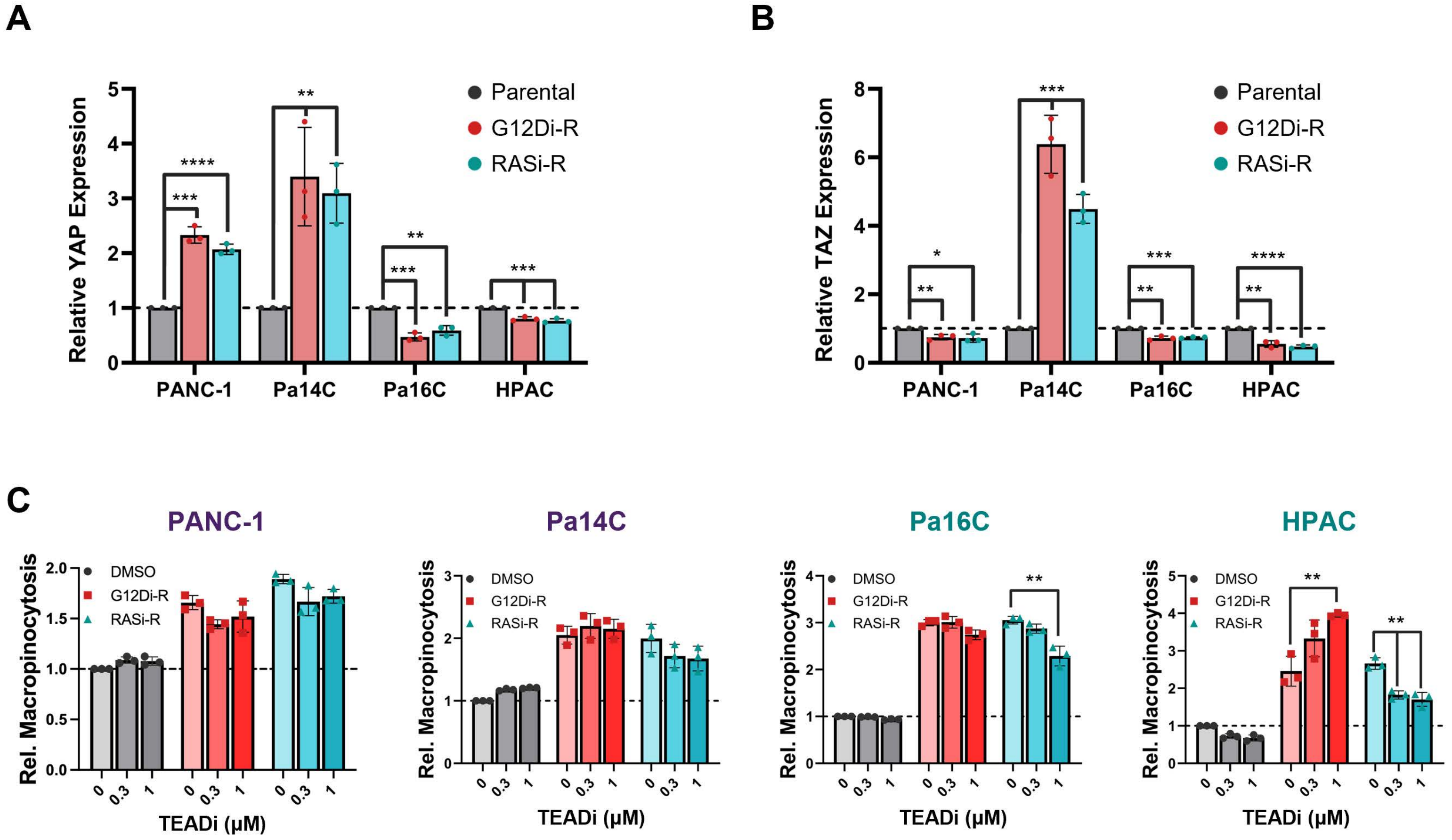

A
B
Relative YAP Expression
Relative TAZ Expression
Parental
G12Di-R
RASi-R
PANC-1
Pa14C
Pa16C
HPAC
C
PANC-1
Pa14C
Pa16C
HPAC
Rel. Macropinocytosis
DMSO
G12Di-R
RASi-R
TEADi (μM)

Supplementary Figure S13. A subset of RASi-resistant PDAC cell lines activates YAP and/or TAZ to upregulate macropinocytosis. (A-B) Densitometry quantitation of total YAP (A) and TAZ (B) from immunoblots shown in Fig. 6C. Data are presented as the mean ± SEM of three independent experiments. * $\rho < 0.05$, ** $\rho < 0.01$, *** $\rho < 0.001$, and **** $\rho < 0.0001$ by the unpaired Student's *t*-test, comparing against each respective parental line. **(C)** Macropinocytosis was measured via flow cytometry in indicated parental, MRTX1133- (G12Di) resistant (R), or RMC-7977- (RASi) resistant (R) KRAS-mutant PDAC cell lines with or without indicated doses of IAG-993 (TEADi). Macropinocytosis was quantified via TMR dextran labeling. Data are presented as the mean ± SEM of three independent experiments. * $\rho < 0.05$, ** $\rho < 0.01$, *** $\rho < 0.001$, and **** $\rho < 0.0001$, by the unpaired Student's *t*-test, comparing each inhibitor treatment against DMSO for each line.
